# NOS2 as a candidate molecular link between air pollutants and depression: an integrated computational and experimental study

**DOI:** 10.3389/fcell.2026.1962251

**Published:** 2026-10-28

**Authors:** Huizheng Zhu, Bei Yin, Jingning Du, Siwei Zhang, Liyuan Su, Shengyu Cai

**Affiliations:** 1 Department of Traditional Chinese Medicine, Shenzhen People’s Hospital (The Second Clinical Medical College, Jinan University, The First Affiliated Hospital, Southern University of Science and Technology), Shenzhen, Guangdong, China; 2 Affiliated Jiangmen Traditional Chinese Medicine Hospital of Jinan University, Jiangmen, Guangdong, China

**Keywords:** air pollution, depression, machine learning, mendelian randomization, network toxicology, nitric oxide synthase 2, single-cell virtual perturbation

## Abstract

**Introduction:**

Studies exploring the molecules and potential mechanisms linking air pollution exposure to depression remain scarce. This study aimed to elucidate the potential regulatory molecules and possible mechanisms associated with air pollutants and depression, thereby providing scientific clues for disease prevention and treatment.

**Methods:**

Seven air pollutants (SO_2_, NO, benzene, CO, O_3_, NO_2_, and toluene) were included. A multilevel research framework was employed. Network toxicology and the Gene Expression Omnibus (GEO) database were used to identify potential targets and pathways of air pollutants; intersection genes were used to construct machine learning diagnostic models, and the optimal model was selected for validation. Mendelian randomization (MR) was applied to assess the genetic causal association between depression and relevant genes. Single-cell virtual perturbation was performed for network perturbation analysis to dissect potential molecular mechanisms. Molecular docking and molecular dynamics simulations were used to evaluate the binding affinity and complex stability between specific pollutants and depression-related genes. Finally, *in vitro* cell experiments were conducted to preliminarily validate the computational findings.

**Results:**

By intersecting air pollution-related targets with the Gene Expression Omnibus depression dataset, nine candidate target genes were identified. Ensemble machine learning analysis suggested that nitric oxide synthase 2 (NOS2) showed expression in depression. Mendelian randomization analysis suggested that elevated NOS2 expression may be associated with depression, supporting a possible genetic causal association between the two. Single-cell virtual perturbation suggested that perturbing NOS2 in microglia could affect inflammation- and oxidative stress-related pathways. Molecular docking predicted that all seven pollutants might bind to NOS2, but the predicted binding energies varied, with benzene and toluene showing relatively higher binding affinity. Molecular dynamics simulations further evaluated the stability of the benzene–and toluene–NOS2 complexes, suggesting that toluene binding to NOS2 was more stable. *In vitro* cell experiments preliminarily suggested that toluene exposure could induce inflammation and oxidative stress in BV2 cells, and changes in NOS2 expression may be involved in this process. However, these results cannot yet prove that NOS2 is a causal mediator of depression, and further functional experiments are needed for validation.

**Discussion:**

Integrating computational prediction, genetic association, network perturbation, and cellular validation, NOS2 may be a candidate molecular node linking air pollution exposure to depression, and its potential mechanism may involve neuroinflammation and oxidative stress. This study provides new hypotheses and theoretical clues for the possible molecular mechanisms linking air pollutants and depression, but the underlying mechanisms still require further experimental investigation.

## Graphical Abstract

**Figure:**
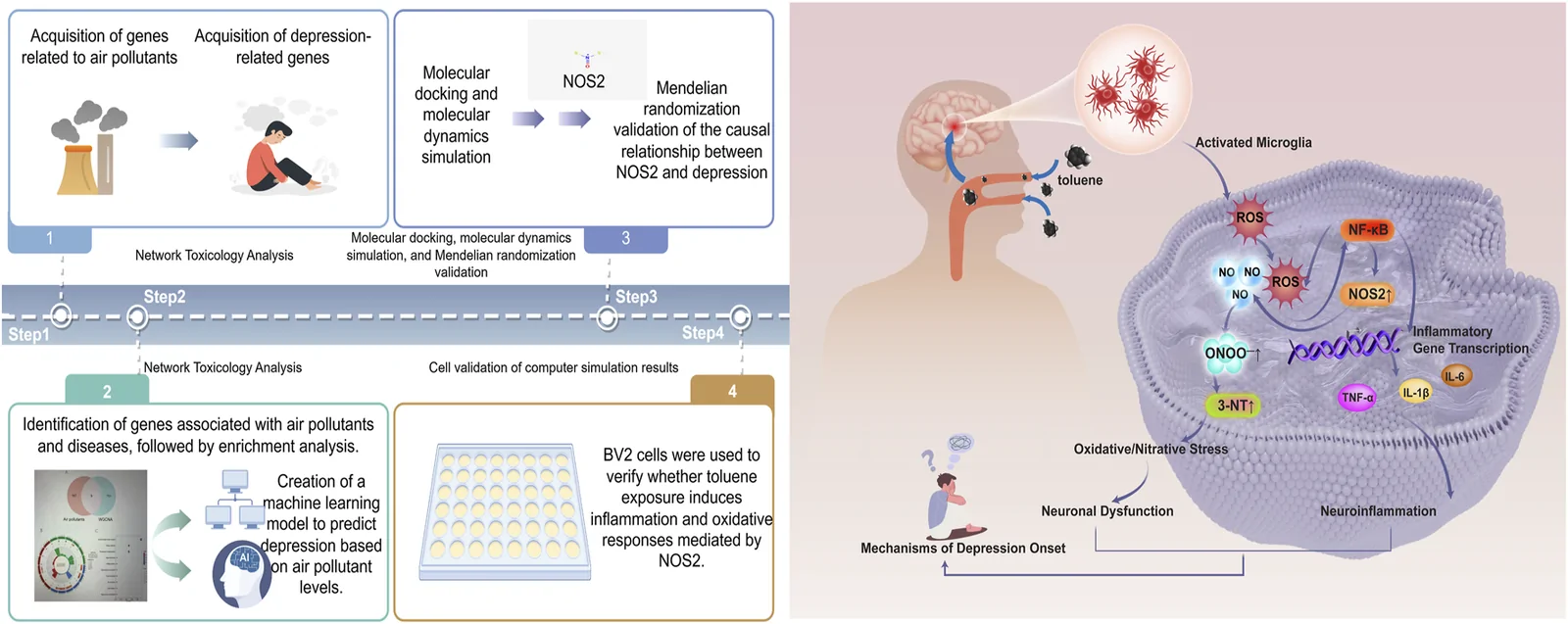

## Introduction

The International Agency for Research on Cancer has classified air pollution as a Group I carcinogen based on evidence of its carcinogenicity in experimental animals and humans (Loomis et al., 2013). According to the 2019 Global Burden of Disease (GBD) study (GBD 2019 Risk Factors Collaborators, 2019), air pollution is the fourth-ranked risk factor for global deaths, accounting for approximately 2.92 million deaths among males and 3.75 million deaths among females. A meta-analysis of 12 studies (61,217 participants) (Zhang et al., 2024) suggested a significant association between indoor air pollution (IAP) exposure and depression (risk ratio (RR) = 1.22, 95% confidence interval: 1.13–1.31). Exposure to IAP from solid fuel use was associated with an increased risk of depression (RR = 1.20). Therefore, understanding the adverse health effects of air pollutants and analyzing their dose-response relationships are critical for developing air pollution prevention strategies, establishing ambient air quality standards, and formulating public health policies.

Depression is a mental disorder with unclear pathological mechanisms and is characterized by substantial and persistent depressed mood, loss of interest, and psychomotor retardation; it is often accompanied by psychiatric and physical comorbidities such as eating or sleep disorders (Gold et al., 2015; Federici et al., 2017). As a highly prevalent mental disorder, depression primarily impairs emotional regulation and is associated with substantial socioeconomic burden, including increased healthcare resource utilization and economic burden on families. The World Health Organization reports that the global average prevalence of depression is 10% and is rising annually. Recent studies (Pan et al., 2025; Yu et al., 2025; Zhao et al., 2025) have primarily evaluated the correlation between air pollution exposure and depression; however, the underlying mechanisms are not well-characterized. Several studies have suggested the involvement of inflammation and oxidative stress (Babadjouni et al., 2017); however, specific molecular and biological processes have not been identified or characterized.

Nitric oxide (NO) is generated from arginine by NO synthases (NOS). NOS2 is an inducible isoform, whereas NOS1 (neuronal NOS) and NOS3 (endothelial NOS) are constitutively expressed at basal levels (Bogdan, 2015). NO is a crucial signaling molecule that plays a significant role in several biological processes, including immune response regulation, apoptosis, vasodilation, angiogenesis, and platelet inhibition (Dillon et al., 2020). NO is involved in multiple physiological functions and is aberrantly expressed in several disorders. Nitric oxide synthase 2 (NOS2) is recognized as a susceptibility gene for inflammatory disorders. Several research studies have reported that the neuroinflammatory pathway is associated with the development, treatment response, and remission of major depressive disorder. Oxidative stress is the predominant driver of inflammatory system dysfunction observed in patients with depression and is caused by excessive NO production driven by NOS2 (inducible nitric oxide synthase) (Wigner et al., 2018). However, the link between air pollutants, NOS2, and depression remains unknown.

In this study, we integrated network toxicology, machine learning, molecular docking, molecular dynamics simulations, Mendelian randomization (MR), and single-cell virtual perturbation to evaluate a potential link between seven common air pollutants (benzene, SO_2_, NO, CO, NO_2_, toluene, and O_3_) and depression. Potential targets and pathways affected by air pollutants were identified by integrating network toxicology with data from the Gene Expression Omnibus (GEO) database. The intersecting genes were used to construct machine learning-based diagnostic models and the optimal model was selected for validation. Mendelian randomization was performed to determine the genetic causal relationship between depression and relevant genes. Single-cell virtual knockout was used to elucidate the underlying molecular mechanisms. Molecular docking and molecular dynamics simulations were used to evaluate the binding affinity between specific pollutants and depression-related genes. *In vitro* experiments were performed to validate the computational findings. Through computational analyses and *in vitro* experiments, this study demonstrates that NOS2 may be a regulatory factor in the relationship between air pollutants and depression. These findings deepen our understanding of the effects of air pollution and provide a scientific basis for mitigating pollution and addressing depression.

## Methods and materials

The workflow of the computer simulation is shown in Figure 1.

**FIGURE 1:**
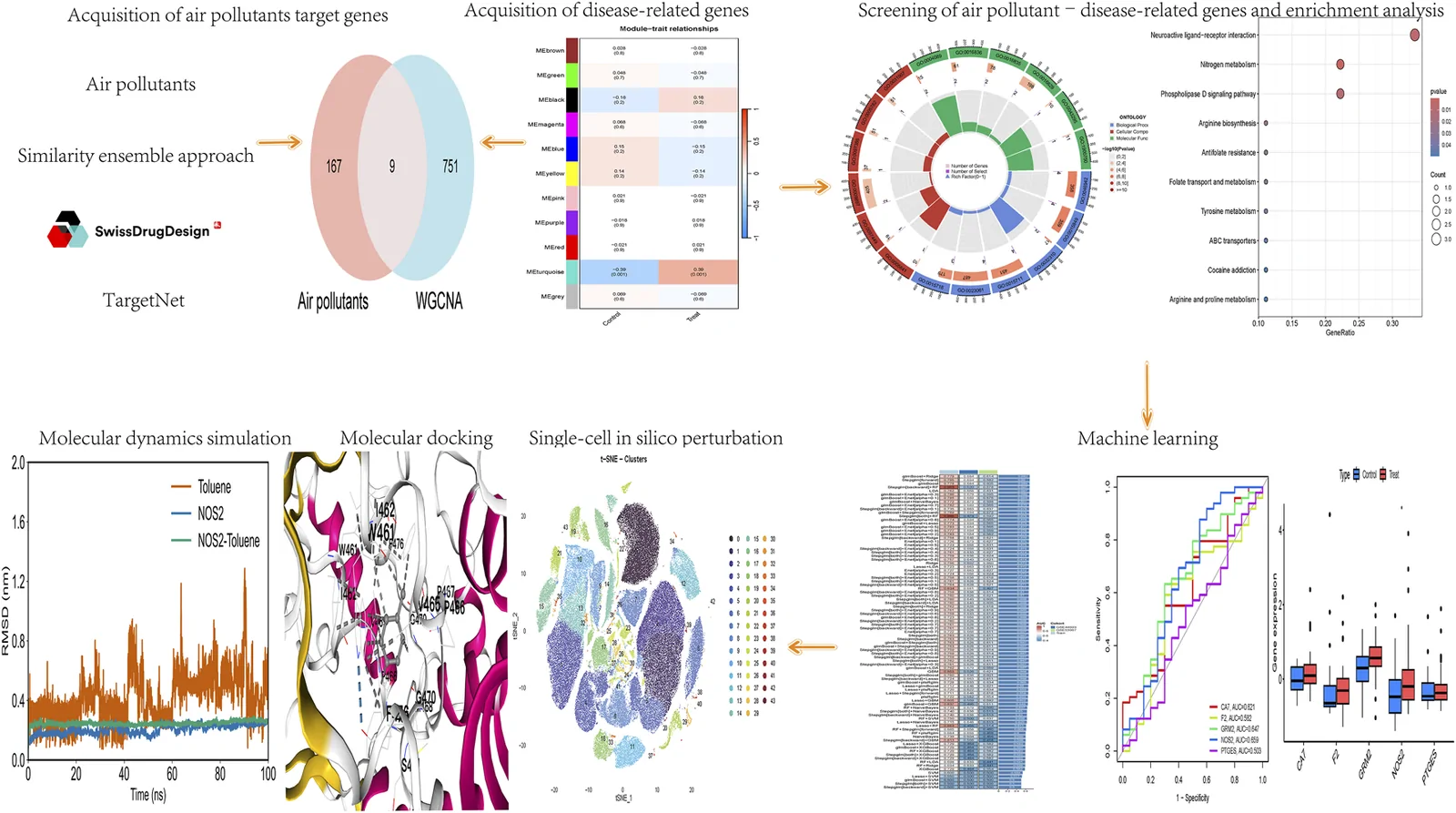
Overview of computer-aided simulation design.

### Identification of target genes related to air pollutants

Human target genes potentially linked to the seven air pollutants were identified using the Target Net (http://targetnet.scbdd.com), Swiss Target Prediction (http://www.swisstargetprediction.ch/), and SEA (https://sea.bkslab.org/) databases. Genetic data from these sources were integrated and compiled into a comprehensive set of target genes for air pollutants.

### Disease-associated transcriptomic data acquisition and sample composition

Processed peripheral blood gene expression data was obtained from the GSE32280 and GSE76826 datasets. All available samples from both datasets were included. The GSE32280 dataset comprised 37 samples, including eight healthy controls, eight pretreatment MDD samples, eight post-treatment MDD samples, eight pretreatment subsyndromal symptomatic depression samples, and five post-treatment subsyndromal symptomatic depression samples. The GSE76826 dataset consisted of 32 samples, including 12 healthy controls, 10 samples collected during a depressive episode, and 10 remission samples.

For the binary analysis, the 20 healthy-control samples were classified as “Control,” whereas the remaining 49 samples were classified as “Treat.” The “Treat” group represented the combined non-control/depression-spectrum cohort, which encompassed pretreatment, post-treatment, depressive-state, remission, MDD, and subsyndromal depression samples. None of the samples were excluded based on the diagnosis, treatment status, or remission status.

GSE32280 was generated from peripheral blood lymphocytes using the Affymetrix Human Genome U133 Plus 2.0 Array (GPL570). The GEO processed data were normalized by robust multiarray average normalization and log2 transformed. GSE76826 was generated from the peripheral blood leukocytes using the Agilent SurePrint G3 Human GE v2 8 × 60-K platform (GPL17077). The original investigators performed background subtraction and 75th percentile per-chip normalization; however,per-gene normalization was not conducted.

### Data integration and batch correction

The input expression matrices were converted to gene-symbol-level matrices before the integration analysis. Genes shared by both datasets were retained, and duplicated gene symbols were averaged using the “avereps” function in the limma package. A total of 17,633 non-empty gene symbols were common to the two input matrices. The matrices were merged by the gene symbol. The cohort/platform-associated batch effects were adjusted using the empirical-Bayes ComBat algorithm. GSE32280 and GSE76826 were treated as separate batches. Expression-distribution boxplots and principal component analysis were used to assess the data before and after batch correction.

### Weighted gene co-expression network analysis

We retained 2,341 genes with an across-sample standard deviation (SD) greater than 0.5 for weighted gene coexpression network analysis (WGCNA). The sample and gene quality were assessed using the “goodSamplesGenes” function. Potential sample outliers were evaluated using average-linkage hierarchical clustering based on Euclidean distances. Static tree cutting was performed with a cut height of 20,000 and a minimum cluster size of 10.

Soft-thresholding powers from 1 to 20 were evaluated using “pickSoftThreshold.” A power of 3 was selected as the first value achieving a scale-free topology fit greater than 0.80. Then, an unsigned adjacency matrix and a topological overlap matrix was constructed. We identified 11 modules, including the gray module, using average-linkage hierarchical clustering and dynamic tree cutting, with a minimum module size of 60, deepSplit = 2, and pamRespectsDendro = FALSE. Modules with eigengene dissimilarity below 0.25 were merged. Module eigengenes were correlated with the correctly matched “Control” and “Treat” labels using Pearson correlation, and Student asymptotic P values were calculated.

### Screening of air pollutant–disease-related genes and enrichment analysis

Venn diagram was constructed to identify the common gene targets for air pollution and depression. The intersecting genes were considered as potential toxicological targets for air pollution-induced depression. To assess the functional biological roles of the intersecting air pollution-related depression genes, gene ontology (GO) functional annotation and Kyoto Encyclopedia of Genes and Genomes (KEGG) enrichment analyses were performed using the “ClusterProfiler,” “Org. Hs. e.g.db” and “Enrichplot” R packages.

The GO analysis was used to determine the enriched biological processes, cellular components, and molecular functions to clarify the primary biological roles of these targets. KEGG enrichment analysis was used to determine the key pathways linked to potential targets of air pollution-induced depression.

### Construction of a machine learning model for screening potential regulatory molecules in air pollution-associated depression

Machine learning was used to evaluate whether the nine candidate genes shared between the air-pollution and depression analyses could distinguish the combined non-control group from healthy controls. The candidate factors were NOS2, HRH4, PNMT, GRM2, PTGES, ABCG2, CA3, F2, and CA7. The training set combined GSE32280 (GSM799723–GSM799758) and GSE76826(GSM2038455–GSM2038486) and contained 69 expression profiles, including 20 healthy-control and 49 non-control profiles. Two independent datasets were used for external evaluation: GSE44593(GSM1087510–GSM1087537) contained 28 samples, including 14 controls and 14 non-control samples, and GSE53987(GSM1304870–GSM1304904) contained 35 samples, including 18 controls and 17 non-control samples.

For data integration, genes shared among the expression matrices were retained, and duplicate gene symbols were averaged using the “avereps” function in the limma package. Cohort-related batch effects were adjusted using the empirical-Bayes ComBat algorithm. The nine candidate genes were then extracted from the batch-adjusted matrix. Expression values in the training dataset were centered and scaled, and the validation data were standardized separately within each validation cohort.

We evaluated a collection of binary classification pipelines using diverse base methods: support vector machines; ridge, Lasso, and elastic-net logistic regression; component-wise gradient boosting with a binomial loss; sparse partial least-squares logistic regression; forward, backward, and bidirectional stepwise logistic regression; classification random forests; gradient boosting machine with a Bernoulli loss; linear discriminant analysis; XGBoost with a binary logistic objective; and naive Bayes classification. Penalized logistic regression models were fitted using the “glmnet” function. Ridge and Lasso corresponded to elastic-net mixing parameters of α = 0 and α = 1, respectively, and additional elastic-net models were evaluated at α values from 0.1 to 0.9. The regularization parameter λ was selected by cross-validation using the minimum cross-validated error. Support vector machines were trained using the e1071 R package, enabled with probability estimation. Component-wise logistic boosting was implemented using mboostglmboost with a binomial family. Sparse partial least-squares logistic regression was implemented using the “plsRglm” function. Classification random forests were fitted using “randomForestSRC” function with 1,000 trees. The gradient-boosting machines used a Bernoulli loss, an interaction depth of 3, a minimum terminal-node size of 10, and a learning rate of 0.001. Linear discriminant analysis was implemented using the caret package; XGBoost was configured using a binary logistic objective; and naive Bayes classification was implemented using the e1071 package.

We evaluated single-method models and two-stage combinations. In a two-stage pipeline, the first algorithm selected candidate predictors and the second algorithm fitted a binary classifier using selected predictors. Feature-selection was conducted using Lasso, component-wise logistic boosting, classification random forest, and stepwise logistic regression methods. Models retaining two or fewer predictors were excluded. The random seed was set to 123 for improving reproducibility. Among the 111 prespecified single-method and two-stage pipelines, 87 qualified for evaluation based on the feature-retention criterion.

Model discrimination was evaluated using the area under the receiver-operating-characteristic curve (AUC) values in the training set and in the GSE44593 and GSE53987datasets. AUC values were calculated using the pROC package. The 95% confidence interval for the AUC of the selected model was estimated by bootstrap resampling. A heatmap displayed the AUC values for the training and validation datasets, whereas the performance was evaluated using the cohort-specific receiver-operating-characteristic curves and confusion matrices.

### Mendelian randomization analysis

A two-sample Mendelian randomization analysis was performed to analyze the association between genetically proxied *NOS2* expression and depression. Summary-level genetic associations with *NOS2* expression were obtained from the eQTLGen Consortium whole-blood eQTL dataset through the Integrative Epidemiology Unit (IEU) OpenGWAS platform (OpenGWAS ID: eqtl-a-ENSG00000007171; Ensembl ID: ENSG00000007171). The eQTLGen resource predominantly included participants with European ancestry. The per-variant sample sizes of the retained NOS2 instruments ranged from 20,989 to 29,285.

Candidate instruments associated with *NOS2* gene expression were selected at a genome-wide significance threshold of P < 5 × 10^−8^. Cis instruments were defined as variants located within 500 kb upstream or downstream of the *NOS2* gene boundaries on GRCh37 (chromosome 17:26,083,792–26,127,525). Eight variants were selected after Linkage Disequilibrium (LD) clumping analysis of 129 variants using an r^2^ threshold of 0.20 within a 100-kb window with the European 1000 Genomes reference panel. For each variant, the proportion of exposure variance was estimated as (R^2 = \beta^2/[\beta^2 + (SE^2\times N)]), and instrument strength was calculated as (F = [R^2/(1-R^2)]\times (N-2)). Variants with F ≤ 10 were excluded to minimize weak-instrument bias.

Depression summary statistics were obtained from the IEU OpenGWAS database (ID: ebi-a-GCST90038650). The dataset consisted of 27,568 cases and 457,030 controls (total N = 484,598) of European ancestry and was mapped to GRCh37. Corresponding outcome associations were extracted for the selected instruments. The exposure and outcome alleles were harmonized using “harmonise_data” function from the Two-Sample MR package (action = 2). Palindromic variants were evaluated using effect-allele-frequency and ambiguous variants were excluded.

The inverse-variance-weighted method was used as the primary estimator. Complementary analyses were performed using MR-Egger regression, weighted-median, simple-mode, and weighted-mode estimators. Heterogeneity was evaluated using the Cochran’s Q statistic. Directional horizontal pleiotropy was assessed using the MR-Egger intercept; global horizontal pleiotropy and outliers were evaluated using MR-PRESSO(Pleiotropy RESidual Sum and Outlier) Leave-one-out analysis was performed to assess whether the estimate was driven by an individual variant.

### Single-cell virtual perturbation

Virtual perturbation analysis of GSE201687, a single-nucleus RNA sequencing dataset of the dorsolateral prefrontal cortex (dlPFC, BA9), was performed using scTenifoldKnk. The GSE201687 dataset consisted of 14 biological samples, including 7 from a depression-like group and 7 from a healthy control group. For between-group comparisons, the microglia were designated as those from the depression-like group, healthy control group, or psychological resilience group. For the scTenifoldKnk analysis, the default settings of the package were as follows: qc_mtThreshold = 0.1, qc_minLSize = 1000, nc_nNet = 10, nc_nCells = 500, nc_nComp = 3, nc_q = 0.9, td_K = 3, td_maxIter = 1000, and td_maxError = 1e-05. Differentially regulated genes were identified using a threshold of FDR <0.05. For quality control, nuclei with fewer than 200 expressed genes and nuclei with a mitochondrial unique molecular identifier (UMI) proportion greater than 25% were removed. Mitochondrial genes were removed from the expression matrix. The cell-level expression regression was used to avoid the influence of cell-state effects on the clustering analysis and cluster-specific marker analysis. To select highly variable genes, the merged expression matrix was subjected to NormalizeData (LogNormalize method; default scale factor = 10,000), followed by FindVariableFeatures (selection.method = “vst”), ScaleData for Z-score normalization, and finally RunPCA was used to compute principal components. Harmony was used for integration because of potential interindividual batch effects. PCA embeddings were corrected using RunHarmony, and Uniform Manifold Approximation and Projection (UMAP) dimensionality reduction and clustering analyses were subsequently performed based on the Harmony reduction.

### Molecular docking

The crystal structure of human inducible nitric oxide synthase (NOS2) was obtained from the Research Collaboratory for Structural Bioinformatics (RCSB) Protein Data Bank (PDB ID: 2NSI; UniProt accession: P35228). The structure of the NOS2 haem-containing oxygenase domain was determined by X-ray crystallography at a resolution of 3.00. Å. The deposited coordinate file contained four protein chains (A–D), with chains A–B and C–D forming two equivalent biological homodimers. Each chain corresponded to NOS2 amino acid residues 74–504, with resolution of residues 83–502.

Molecular docking was performed using the CB-Dock web server (Cavity-detection guided blind docking; https://cadd.labshare.cn/cb-dock/accessed). The original 2NSI PDB file was submitted as the receptor. According to the default CB-Dock workflow, all heteroatoms, including crystallographic water molecules, sulfate ions, cocrystallized ethylisothiourea inhibitor, heme, and tetrahydrobiopterin, were removed automatically before docking.

The chemical structures of the tested pollutants were obtained from PubChem. The submitted compounds were benzene (CID 241), toluene (CID 1140), carbon monoxide (CID 281), nitric oxide (CID 145068), nitrogen dioxide (CID 3032552), sulfur dioxide (CID 1119), and ozone (CID 24823). CB-Dock converted the submitted Structure-Data File (SDF) files using Open Babel as part of its automated workflow. CB-Dock first detected putative binding cavities using its protein-surface curvature-based cavity-detection procedure. The candidate cavities were ranked according to the cavity sizes, after which the server automatically calculated the center coordinates and dimensions of a ligand-specific docking box for each cavity. The default setting of five candidate cavities was used. Molecular docking was then performed independently within each predicted cavity using AutoDock Vina version 1.1.2. The cavities were ranked according to their best Vina scores, expressed in kcal/mol, and the first-ranked conformation with the most negative Vina score was selected as the representative binding pose.

### Molecular dynamics simulation

Molecular dynamics simulations were conducted for the complexes of NOS2 protein with benzene and toluene using the Gromacs 2024.4 software. Amber14sb was selected as the protein force field, with Gaff2 as the ligand force field. TIP4P water model was used to add solvent to the protein-ligand system and establish a water box with a periodic boundary of 1.2 nm. The Particle Mesh Electrostatics method was used to calculate long-range electrostatic interactions. The Monte Carlo ion placement method was used to introduce appropriate number of sodium and chloride ions to neutralize the overall system charge. Prior to the formal simulation, system energy minimization and equilibration were performed by executing the following two steps: (1) Energy minimization was achieved for each system using the steepest descent algorithm for 50,000 steps (Stop minimization when the maximum force <1000 kJ/mol/nm); (2) Each system was preequilibrated for 50,000 steps at a step size of 2 fs while maintaining constant particle number, volume, and temperature (310 K). After system energy minimization and equilibration, molecular dynamics simulations were performed for 100 ns at a step size of 2 fs without any constraints, with structural coordinates being saved every 10 ps.

Finally, we analyzed the root mean square deviation (RMSD), root mean square fluctuation (RMSF), radius of gyration (Rg), solvent accessible surface area (SASA), as well as the relative free energy distribution and the complex structures were compared at five time points: 0, 25, 50, 75, and 100 ns, in the molecular dynamics simulation trajectories of NOS2 protein complexes with benzene and toluene. Furthermore, we calculated the average binding free energy between the protein and ligand using the Molecular Mechanics/Generalized Born Surface Area (MM/GBSA) method.

### Cell culture, cell counting Kit-8(CCK8) and lactate dehydrogenase (LDH) assays

BV2 cells were purchased from the Cell Bank of the Chinese Academy of Sciences (SCSP-5208) and used for experimentation at passages P5–P10 after confirming absence of *mycoplasma* contamination. Cells were removed from the liquid nitrogen tank, quickly thawed in a 37 °C water bath, and transferred to a centrifuge tube inside a biosafety cabinet. They were centrifuged at 1000 rpm for 5 min. The supernatant was removed and the cells were resuspended in complete culture medium. The cell suspension was transferred to a culture flask and incubated in a 5% CO_2_ incubator. When the cell density reached 80%–90%, the cells were passaged by trypsinization. The culture medium was removed and the cells were incubated with 1 mL of trypsin. The plate was gently shaken and the cells were observed under an inverted microscope. When the cells began to detach, fresh culture medium was added to terminate digestion. The cells were transferred to a centrifuge tube and centrifuged at 1000 rpm for 5 min. The supernatant was discarded and the cells were resuspended in fresh complete culture medium. The cell suspension was passaged into a new flask at a 1:2 ratio. The cells were incubated with fresh BV2 cell complete medium that consisted of Dulbecco’s Modified Eagle Medium (DMEM) with 10% Fetal Bovine Serum (FBS) and 1% antibiotics (100 U/mL penicillin, 100 μg/mL streptomycin).

A 200 mM stock solution of toluene was prepared in Dimethyl Sulphoxide (DMSO). An aliquot of the stock solution was then added to the complete cell culture medium and mixed thoroughly to obtain working solutions in which toluene was dispersed in the medium (final concentrations: 20, 50, and 100 μM; final DMSO concentration: <0.05%). The plate was covered with its lid, and the edges of the lid were wrapped with Parafilm. Several holes were then punctured with a needle to minimize toluene evaporation while not blocking CO_2_ gas exchange. Toluene concentrations reflected theoretical nominal values.

The cells were seeded overnight in a 96-well plate (1 × 10^4 cells per well). The next day, different concentrations of toluene (20, 50, 100 μM) were added to each well. After 24 and 48 h of incubation, 10 μL of CCK8 solution was added to each well and the plates were further incubated for 1–4 h. Then, optical density (OD) values were obtained at 450 nm using a microplate reader and the cell viability curve was plotted.

LDH assay was performed according to the kit instructions. In a 96-well plate, 1 × 10^4^ cells were seeded per well with a volume of 100 μL and incubated overnight. The next day, different concentrations of toluene were added as described above and the cells were further incubated for 24 and 48 h. Subsequently, LDH levels were measured according to the LDH Cytotoxicity Detection Kit instructions. Briefly, 100 μL of LDH detection working solution was added to each well and mixed on a horizontal shaker at room temperature in the dark for 30 min. Then, 20 μL of stop solution was added to each well. The absorbance was measured at 450 nm. Cytotoxicity or cell death rate was calculated as follows: Sample group absorbance = A_450_ sample - A_450_ sample background; Total LDH group absorbance = A_450_ total LDH - A_450_ total LDH background; Untreated control group absorbance = A_450_ untreated control - A_450_ blank control background; Cytotoxicity or cell death rate = (Sample group absorbance - Untreated control group absorbance)/(Total LDH group absorbance - Untreated control group absorbance) × 100%.

### Real-time quantitative polymerase chain reaction (RT-qPCR)

RT-qPCR was performed to determine the levels of NOS2, TNF-α, IL-1β, IL-6, and NF-κB p65 transcripts. Briefly, mRNA was prepared from the cells and reverse transcribed. The cDNA was then subjected to quantitative fluorescent PCR amplification. Relative gene expression was determined using the 2^−ΔΔCt^ method. The qPCR primers were synthesized by Sangon Biotech (Shanghai). The primer information is shown in Table 1.

**TABLE 1:** Primer information.

Gene	Sequence (5′-3′)
NOS2-mF	GTT​CTC​AGC​CCA​ACA​ATA​CAA​GA
NOS2-mR	GTG​GAC​GGG​TCG​ATG​TCA​C
TNF-α-mF	CTG​AAC​TTC​GGG​GTG​ATC​GG
TNF-α-mR	GGC​TTG​TCA​CTC​GAA​TTT​TGA​GA
IL-1β-mF	TGC​CAC​CTT​TTG​ACA​GTG​ATG
IL-1β-mR	TGA​TGT​GCT​GCT​GCG​AGA​TT
IL-6-mF	TAG​TCC​TTC​CTA​CCC​CAA​TTT​CC
IL-6-mR	TTG​GTC​CTT​AGC​CAC​TCC​TTC
NF-κb p65-mF	GGT​ATG​GCT​ACT​CGA​ACT​ACG​G
NF-κb p65-mR	TTT​CCT​TCT​CAG​GGA​GAG​TCA​G
GAPDH-mF	GGT​GAA​GGT​CGG​TGT​GAA​CG
GAPDH-mR	CTC​GCT​CCT​GGA​AGA​TGG​TG

### Western blot

Western blotting was used to detect the protein levels of NOS2 in cells. The protein samples were prepared from cells using the radioimmunoprecipitation assay buffer lysis buffer with 1 mM Phenylmethylsulfonyl Fluoride. The cells were lysed on ice for 30 min, with shaking every 10 min to ensure complete lysis. The lysate samples were then centrifuged at 12,000 × g for 10 min at 4 °C, and the supernatant was collected. The protein extracts were quantified using the bicinchoninic acid assay. Equal amounts of protein samples (25 μg of protein per lane) were separated using sodium dodecyl sulfate-polyacrylamide gel electrophoresis and transferred onto polyvinylidene fluoride membranes. After blocking, the blots was probed with the following primary antibodies: anti-NOS2 antibody (1:3,000 dilution; Cat. No. WL0992a) and anti-GAPDH rabbit polyclonal antibody (1:10,000 dilution; Cat. No. 10494-1-AP). Subsequently, after incubation with the HRP-conjugated secondary antibody, the blots were developed with ECL and the relative level of NOS2 protein was estimated from the protein bands.

### Nitrite content testing

The supernatant was collected after treating the cells for estimating nitrite levels. Concurrently, Griess Reagents I and II were equilibrated to room temperature. Standard solutions ranging from 0 to 100 µM (0, 1, 2, 5, 10, 20, 40, 60, 100 µM) were prepared in DMEM +10% FBS. In a 96-well plate, 50 µL of the standards and samples (supernatant) were added into each well. Then, 50 µL of Griess Reagent I was added into each well, followed by 50 µL of Griess Reagent II. Subsequently, the absorbance was measured at 540 nm.

### Flow cytometry detection of reactive oxygen species (ROS) levels

The cell suspensions were harvested into a 1.5-mL centrifuge tube and washed twice with 1× phosphate buffered saline (PBS) at 1000 rpm for 5 min. After discarding the supernatant, the cels were resuspended in 500 μL of 1× PBS. Subsequently, the ROS probe (Cat. No. E004-1-1) was added to a final concentration of 10 µM. The cells were incubated at 37 °C for 1 h, with gentle inversion every 3–5 min to ensure thorough contact between the probe and the cells. The ROS probe-labeled single-cell suspensions were centrifuged at 1000 rpm for 5 min. After washing twice with PBS, the cell pellet was resuspended in PBS and analyzed by flow cytometry under fluorescein isothiocyanate fluorescence detection parameters. Flow cytometric gating strategy: the total cell population (P1) was first gated on an FSC-A versus SSC-A plot; ROS signal was then analyzed in the dichlorodihydrofluorescein fluorescence channel using a histogram or scatter plot.

### Immunofluorescence

Cell crawling assay: The cells were seeded in a 24-well plate (precoated with slides) and allowed to adhere. Then, the cells were treated with toluene for 24 h. After treatment, the culture medium was removed and washed once with PBS. The cells were fixed with 4% paraformaldehyde at room temperature for 20 min. After washing twice with PBS, cells were permeabilized with 0.1% Triton X-100 (diluted in PBS) at room temperature for 20 min. Then, cells were incubated with blocking solution (3% Bovine Serum Albumin in PBS) at room temperature for 1 h. Subsequently, they were incubated with the primary antibody (diluted in blocking solution) overnight at 4 °C. After washing three times with PBS, they were incubated with a fluorescence-tagged secondary antibody (diluted in blocking solution) at room temperature for 30 min. Then, after washing three times with PBS, the cells were mounted with the mounting medium containing 4′,6-diamidino-2-phenylindole, and observed and photographed under a laser confocal microscope. Images were analyzed using the ImageJ software to estimate the mean fluorescence intensity.

### Statistical analysis

Data are presented as mean ± SD. Between-group comparisons were performed using one-way Analysis of Variance (ANOVA). Shapiro–Wilk test was used to assess normality. Levene’s test was used to assess homogeneity of variance. When data met the assumptions of normality and homogeneity of variance, Tukey’s *post hoc* test was used for multiple comparisons. When variance was heterogenous, Welch’s ANOVA followed by the Games–Howell or Dunnett’s T3 test was applied. When normality was not met, the Kruskal–Wallis test followed by Dunn’s *post hoc* test was used. A two-tailed P < 0.05 was considered statistically significant. Asterisks (* or **) indicate significant (P < 0.05) or highly significant (P < 0.01) differences compared with the control group; hash symbols (# or ##) indicate significant (P < 0.05) or highly significant (P < 0.01) differences compared with the 50 μM toluene group. N = 3 refers to technical replicates. Statistical analyses and graphing were performed using GraphPad Prism 5 (GraphPad Software, San Diego, CA, USA).

## Results

### Acquisition of genes related to air pollutants

We identified 176 potential target genes related to seven air pollutants using the Target Net, Swiss Target Prediction, and SEA databases. The specific content of air pollution targets is presented in Supplementary Material.

### Acquisition of disease-related genes

The GSE32280 and GSE76826 databases were merged and batch effects were eliminated. Figure 2B shows data after processing batch effects. The processed data were more uniform compared to the non-processed data in Figure 2A. PCA analysis was then conducted to verify the batch effects. As shown in Figure 2C, there was no intersection between the two datasets before processing batch effects. After processing batch effects, there was some intersection between the two datasets, as shown in Figure 2D. After handling the batch effect, WGCNA was performed to allocate coexpressed genes. The “average correlation” and “Pearson correlation value” were estimated and the samples were clustered into datasets, except for outliers (Figure 2E). We selected a soft-threshold power of β = 2 based on a scale independence greater than 0.8, ensuring formation of a significant scale-free biological network (Figure 2F). Hierarchical clustering analysis and dynamic tree cutting identified 11 modules (Figures 2G,H). Correlation analysis of module traits demonstrated a significant association between the turquoise module and depression (Figure 2I). Therefore, genes in the turquoise module were selected for further analysis. To determine the significance of each module, gene significance was calculated to measure the association between genes and sample traits. The correlation between traits was most significant in the turquoise module (Figure 2J). A total of 760 genes were identified in the turquoise module; the scatter diagram shows a strong relevance between these genes and depression (Figure 2K).

**FIGURE 2:**
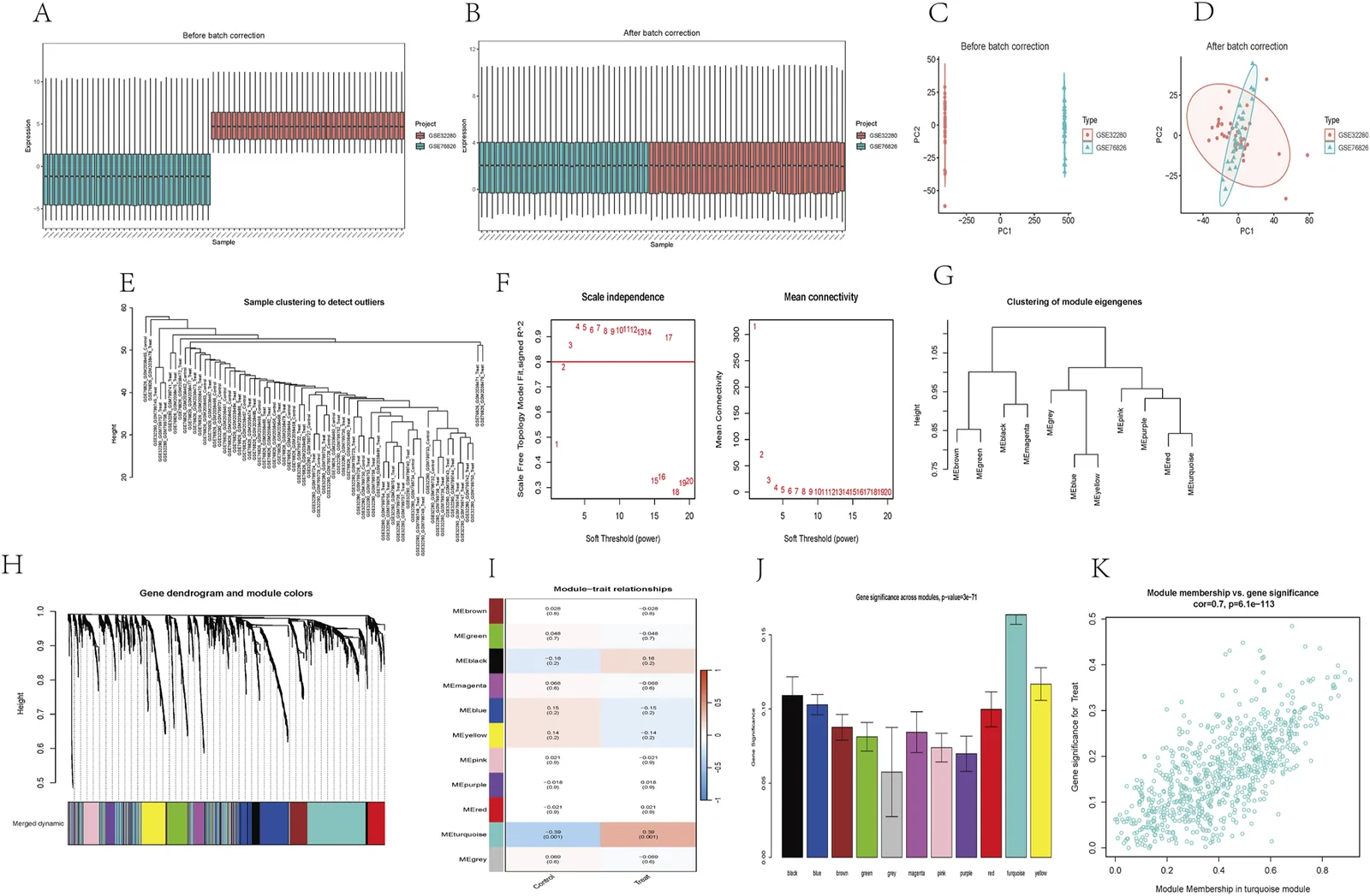
Construction of a weighted co-expression network for depression-related datasets. **(A)** Boxplots before batch effect correction. **(B)** Boxplots after batch effect correction. **(C)** Principal component analysis (PCA) before batch effect correction. **(D)** PCA after batch effect correction. **(E)** Sample clustering tree. **(F)** Analysis of network topology for various soft-thresholding powers. **(G)** Gene clustering dendrogram. **(H)** Gene dendrogram and module assignment based on clustering with different indicators. **(I)** Module–trait relationships. **(J)** Distribution of mean gene significance and associated error within depression-related modules. **(K)** Scatterplot of gene significance versus module membership in the turquoise module.

### Identification of genes associated with air pollutants and diseases, followed by enrichment analysis

Intersection analysis of 176 air pollution targets and 760 depression targets derived from weighted coexpression network analysis identified the following nine genes: *NOS2*, histamine receptor H4 (*HRH4*), phenylethanolamine N-methyltransferase (*PNMT*), glutamate metabotropic receptor 2 (*GRM2*), prostaglandin E synthase (*PTGES*), ATP binding cassette subfamily G member 2 (*ABCG2*), carbonic anhydrase 3 (*CA3*), coagulation factor II (prothrombin) (*F2*), and carbonic anhydrase 7 (*CA7*) (Figure 3A). Functional enrichment analysis of these nine target genes showed that the GO biological functions were mainly enriched in carboxylic acid transport, organic acid transport, the external surface of the plasma membrane, nuclear envelope lumen, carbonate dehydratase activity, and hydrolyase activity (Figure 3B). KEGG analysis showed enrichment of pathways associated with neuroactive ligand–receptor interaction and nitrogen metabolism (Figure 3C).

**FIGURE 3:**
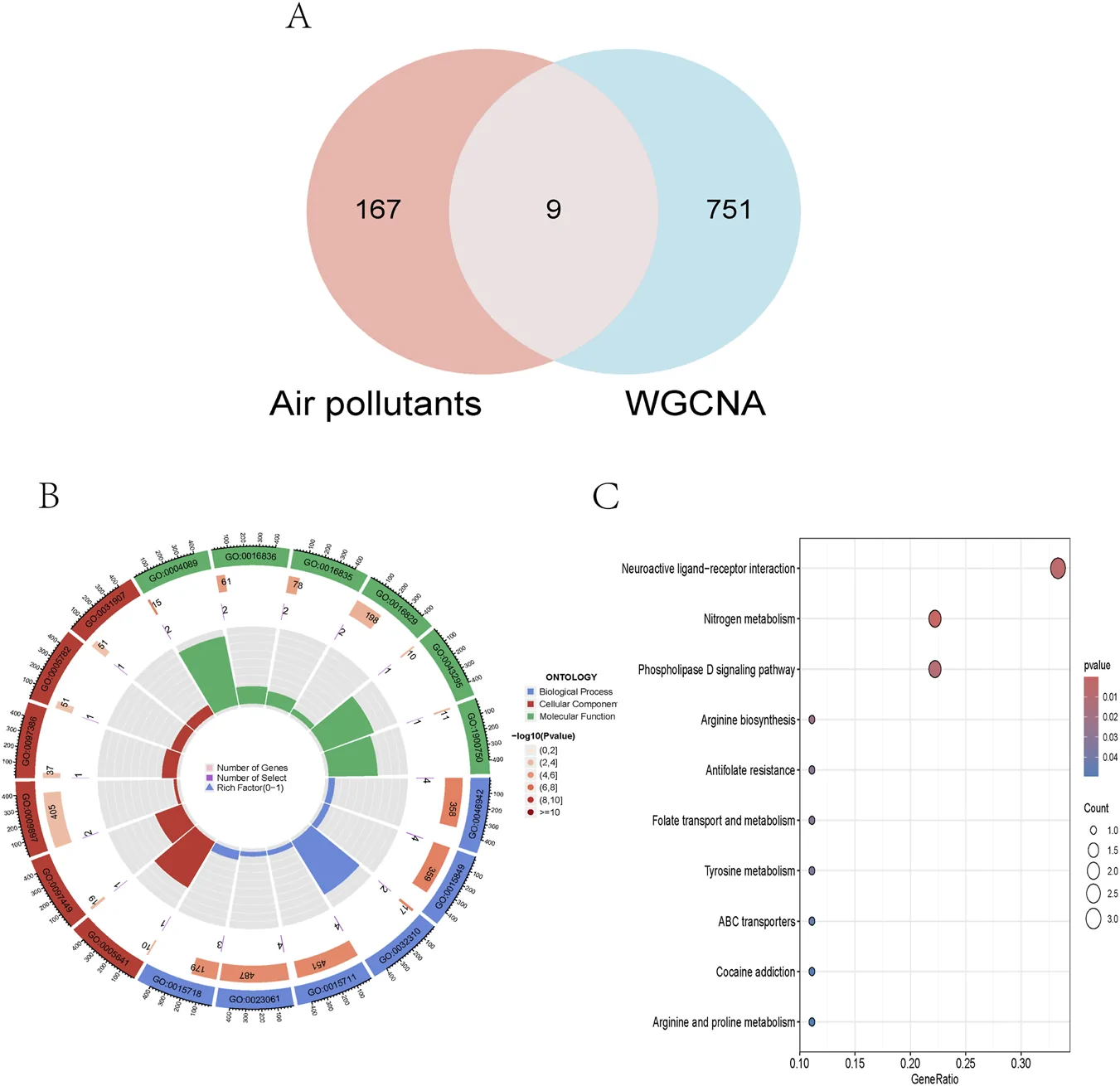
Identification of genes associated with air pollutants and diseases, followed by enrichment analysis. **(A)** Venn diagram displays intersecting genes. **(B)** GO Enrichment Analysis of Targets Related to Air Pollutants and Depression. **(C)** KEGG Enrichment Analysis of Depression Targets Related to Air Pollutants.

### Construction of a machine learning model for depression screening based on air pollutant levels

Integrated machine learning systems were comprehensively assessed using distinct algorithms, and the glmBoost + Ridge model was identified as the top performer, with an average AUC index of 0.692. Among all the generated models, this model was ranked highest and was found to focus on the nine intersecting target genes related to air pollution and depression (Figure 4A). However, this AUC index was only modest.

**FIGURE 4:**
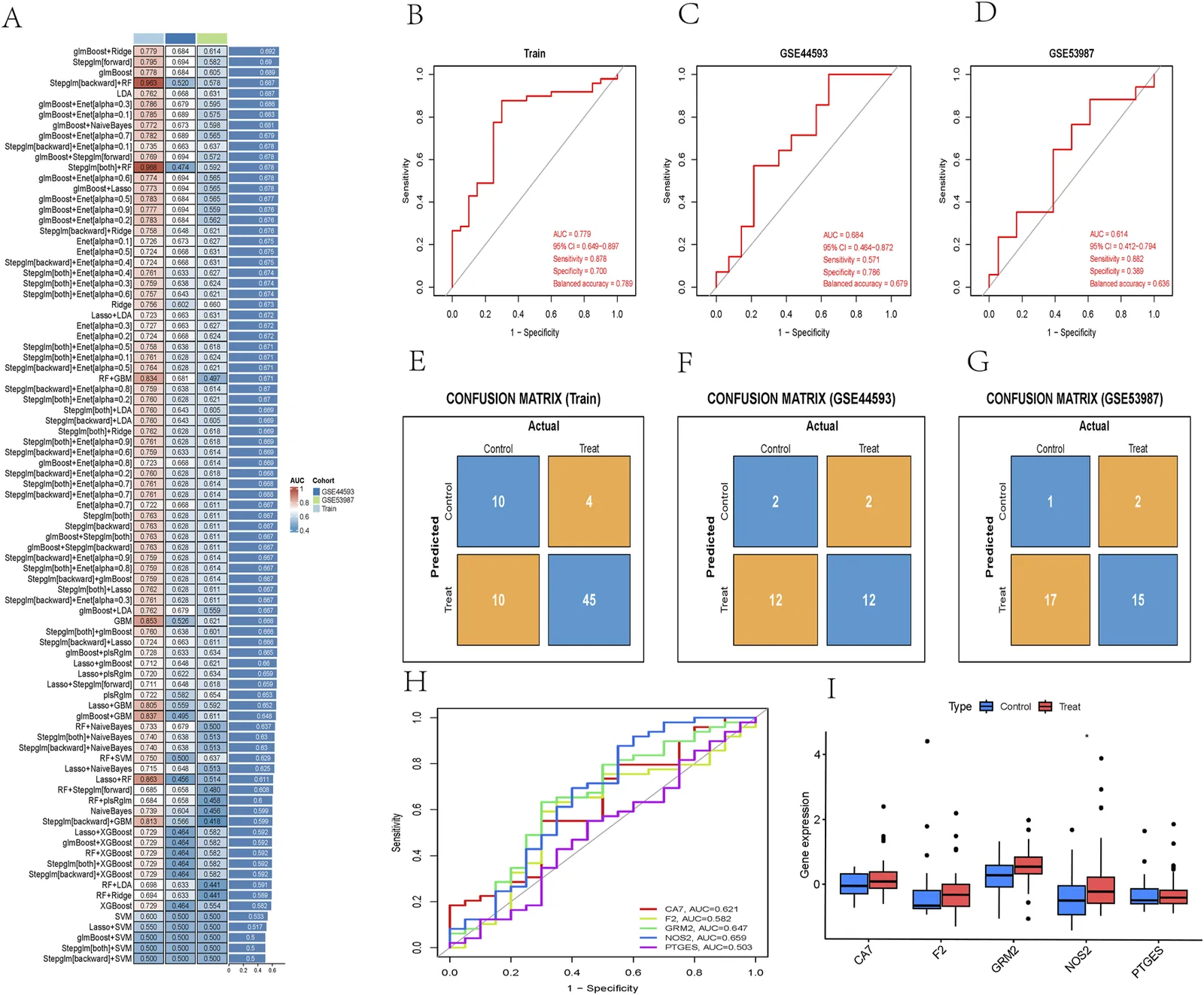
Construction of a machine learning model for depression screening based on air pollutant levels. **(A)** The AUC metric was used to evaluate the performance of machine learning models constructed by integrating multiple algorithms. **(B)**. ROC curve for the training dataset. **(C)** ROC analysis for dataset GSE44593. **(D)** ROC curve of GSE53987. **(E)** The confusion matrix of the training set. **(F)** The confusion matrix of the GSE44593. **(G)** The confusion matrix of the GSE53987. **(H)** ROC curves of 5 key target genes (CA7, F2, GRM2, NOS2, PTGES). **(I)** Differential expression analysis of 5 key target genes (CA7, F2, GRM2, NOS2, PTGES).

### Development and validation of a classification model

We constructed a classification model using the glmBoost + Ridge method. The training set comprised two previously utilized depression datasets: GSE32280 and GSE76826. Based on the AUC values, the model showed significant predictive value (Figure 4B). The model’s reliability and generalizability were assessed by validating two separate external depression datasets, GSE44593 and GSE53987, as illustrated in Figures 4C,D. Then, confusion matrices were used to evaluate the model performance of the training and validation sets, as shown in Figures 4E–G. After screening using the glmBoost + Ridge model method, *CA7* (AUC = 0.621), *F2* (AUC = 0.582), *GRM2* (AUC = 0.647), *NOS2* (AUC = 0.659), and *PTGES* (AUC = 0.503) were selected for further analysis. Among these five genes, *NOS2* showed the highest AUC value (Figure 4H). Appendix 1 reports the AUCs with 95% CIs, sensitivity, specificity, and balanced accuracy for the five genes. Differential expression analysis of the five genes showed that *NOS2* was significantly upregulated in depression (Figure 4I). These findings suggest that *NOS2* may play a key regulatory role linking air pollution with depression. These findings suggest that NOS2 may play a key bridging role between air pollution and depression.

### Mendelian randomization analysis

Integrated Mendelian randomization analysis shows that NOS2 is a key factor mediating the association between air pollution and depression (IVW: odds ratio [OR] = 1.004, 95% confidence interval [CI): 1.000–1.008, P = 0.043). Sensitivity analyses—including leave-one-out, heterogeneity, and pleiotropy assessments—demonstrated that the findings were robust and not influenced by any single nucleotide polymorphism heterogeneity among instrumental variables, or horizontal pleiotropy (Table 2; Figure 5).

**TABLE 2:** Mendelian randomization analysis.

Exposure	Method	nSNP	Beta	Se	P	OR	LCI95	UCI95	Q_pval	Presso	Ple
​	MR egger	8	0.005	0.005	0.379	1.005	0.995	1.016	0.160	0.302	0.811
​	Weighted median	8	0.003	0.002	0.172	1.003	0.999	1.008	​	0.302	0.811
NOS2	Inverse variance weighted	8	0.004	0.002	0.043	1.004	1.000	1.008	0.228	0.302	0.811
​	Simple mode	8	0.001	0.004	0.740	1.001	0.993	1.010	​	0.302	0.811
​	Weighted mode	8	0.003	0.002	0.271	1.003	0.998	1.008	​	0.302	0.811

The table summarizes the results from Mendelian randomization (MR) analyses, reporting for each association the exposure, outcome, analytical method, number of instrumental single-nucleotide polymorphisms (nSNP), beta coefficient (Beta) with its standard error (Se), p-value (P), odds ratio (OR) and its 95% confidence interval (LCI95, UCI95). The Q-test p-value (Q_pval) assesses heterogeneity across genetic instruments. The MR-PRESSO, global test result (Presso) and the pleiotropy test p-value (Ple) are provided to evaluate horizontal pleiotropy. The direction and magnitude of the causal effect are represented by the beta coefficient and OR, while the 95% CI, reflects the precision of each estimate.

**FIGURE 5:**
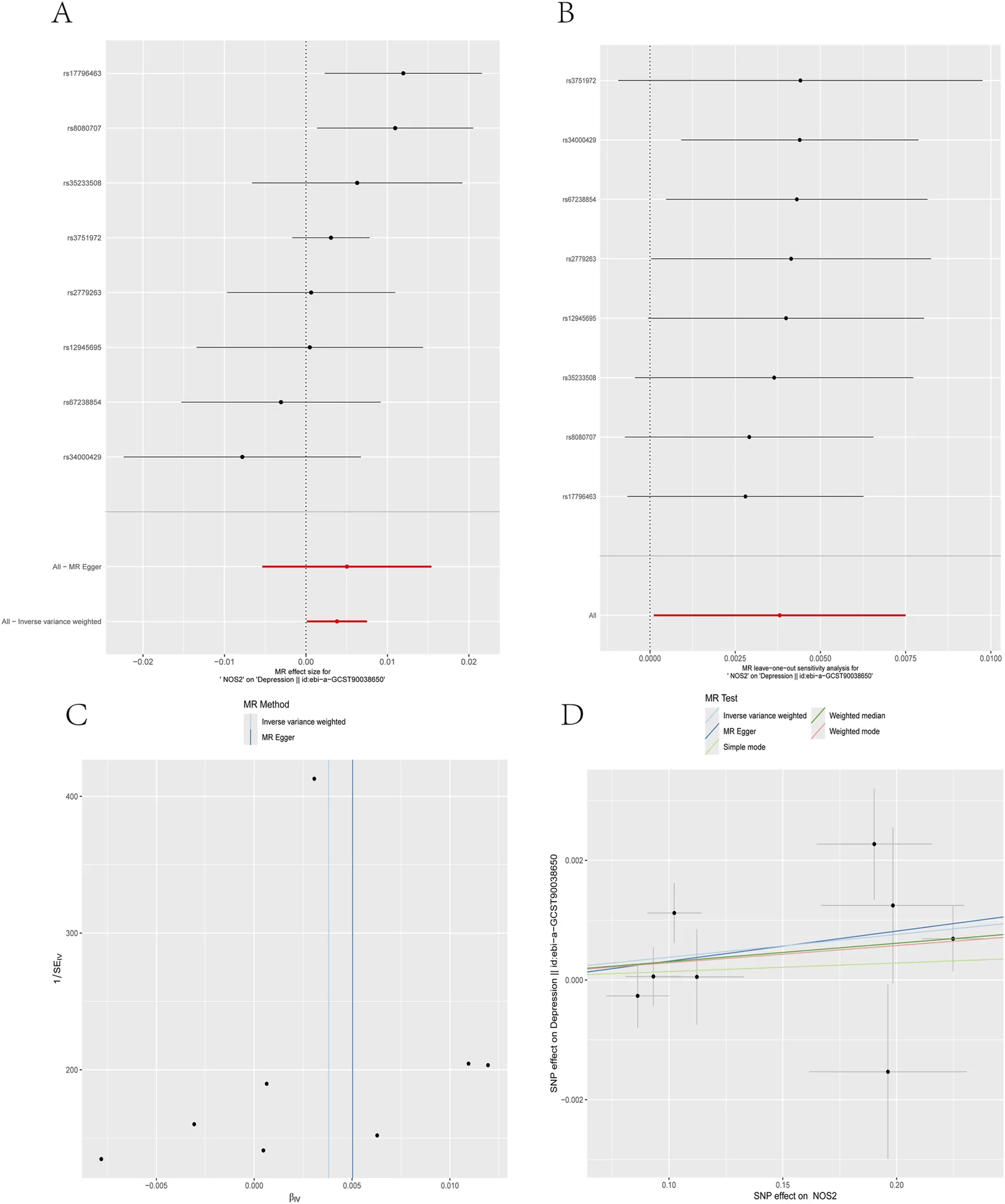
Mendelian randomization (MR) analysis assessing the causal relationship between NOS2 expression and depression. **(A)** Forest plot showing the causal effect estimates of each SNP on depression based on different MR methods, including inverse variance weighted and MR-Egger. **(B)** Leave-one-out analysis plot demonstrating the robustness of the MR results, indicating that the causal association was not driven by any single SNP. **(C)** Funnel plot evaluating heterogeneity and potential horizontal pleiotropy among the instrumental variables; the symmetrical distribution of points suggests the absence of directional bias. **(D)** Scatter plot illustrating the relationship between SNP effects on NOS2 expression (x-axis) and depression risk (y-axis), where regression lines represent causal estimates derived from various MR methods (IVW, MR-Egger, weighted median, weighted mode, and simple mode).

### Single-cell virtual perturbation results

After quality control of the GSE201687 single-cell dataset, t-distributed stochastic neighbor embedding (t-SNE) dimensionality reduction was used to visualize the clustering results, and 43 cell subpopulations were identified (Figure 6A). Figure 6B shows distribution of the depressive and healthy groups in the t-SNE space. Cells from the two groups aligned well across major cell populations, thereby demonstrating effective batch effect correction (e.g., Harmony integration) and comparability between groups. Based on canonical marker genes, the cell populations were annotated into nine major cell types: astrocytes, endothelial cells (Ec), ependymal cells, fibroblasts, gamma-aminobutyric acid-ergic neurons, microglia, neurons, oligodendrocytes, and oligodendrocyte precursor cells (Figure 6C). Figure 6D shows the expression of nitric oxide synthase 2 (NOS2) across different cell types; NOS2 expression was enriched in the microglia. Figure 6E displays canonical marker genes for each cell type (e.g., C-X3-C motif chemokine receptor 1 [CX3CR1] and transmembrane protein 119 [TMEM119] for microglia; glial fibrillary acidic protein [GFAP] and aquaporin 4 [AQP4] for astrocytes), thereby validating the accuracy and reliability of the cell type annotations in Figure 6C. Figure 6F shows the top 20 most significantly differentially regulated genes after virtual perturbation of NOS2 (ranked by log2 fold change). This included Nance–Horan syndrome–like 1 (NHSL1), brain-specific angiogenesis inhibitor 1-associated protein 2-like 2 (BAIAP2L2), versican (VCAN), carboxypeptidase Z (CPZ), serpin family E member 2 (SERPINE2), transglutaminase 2 (TGM2), and serine/threonine kinase 32A (STK32A). The volcano plot displays overall distribution of all the differentially regulated genes; red dots represent significantly differentially regulated genes (false discovery rate [FDR] < 0.05), and the dashed line indicates the significance threshold. Genes with significant expression changes (e.g., BAIAP2L2, VCAN, and NHSL1) are labeled, demonstrating the significance of the virtual knockout effect (Figure 6G). GO enrichment analysis by category showed significantly enriched terms. The enriched GO terms were as follows─ Biological Process: response to oxidative stress, inflammatory response, cellular response to toxic substances, and cellular oxidant detoxification; Cellular components: mainly extracellular matrix, external encapsulating structure, plasma membrane, and secretory granules; Molecular Function: mainly protein serine/threonine kinase activity, antioxidant activity, peroxidase activity, and mitogen-activated protein kinase (MAPK) kinase activity (H). KEGG pathway enrichment analysis revealed that differentially regulated genes were significantly enriched in signaling pathways such as phosphoinositide 3-kinase (PI3K)-Akt, MAPK, oxidative phosphorylation, mammalian target of rapamycin (mTOR), necroptosis, tumor necrosis factor (TNF), nuclear factor kappa-light-chain-enhancer of activated B cells (NF-κB), and interleukin 17 (IL-17) (Figure 6I).

**FIGURE 6:**
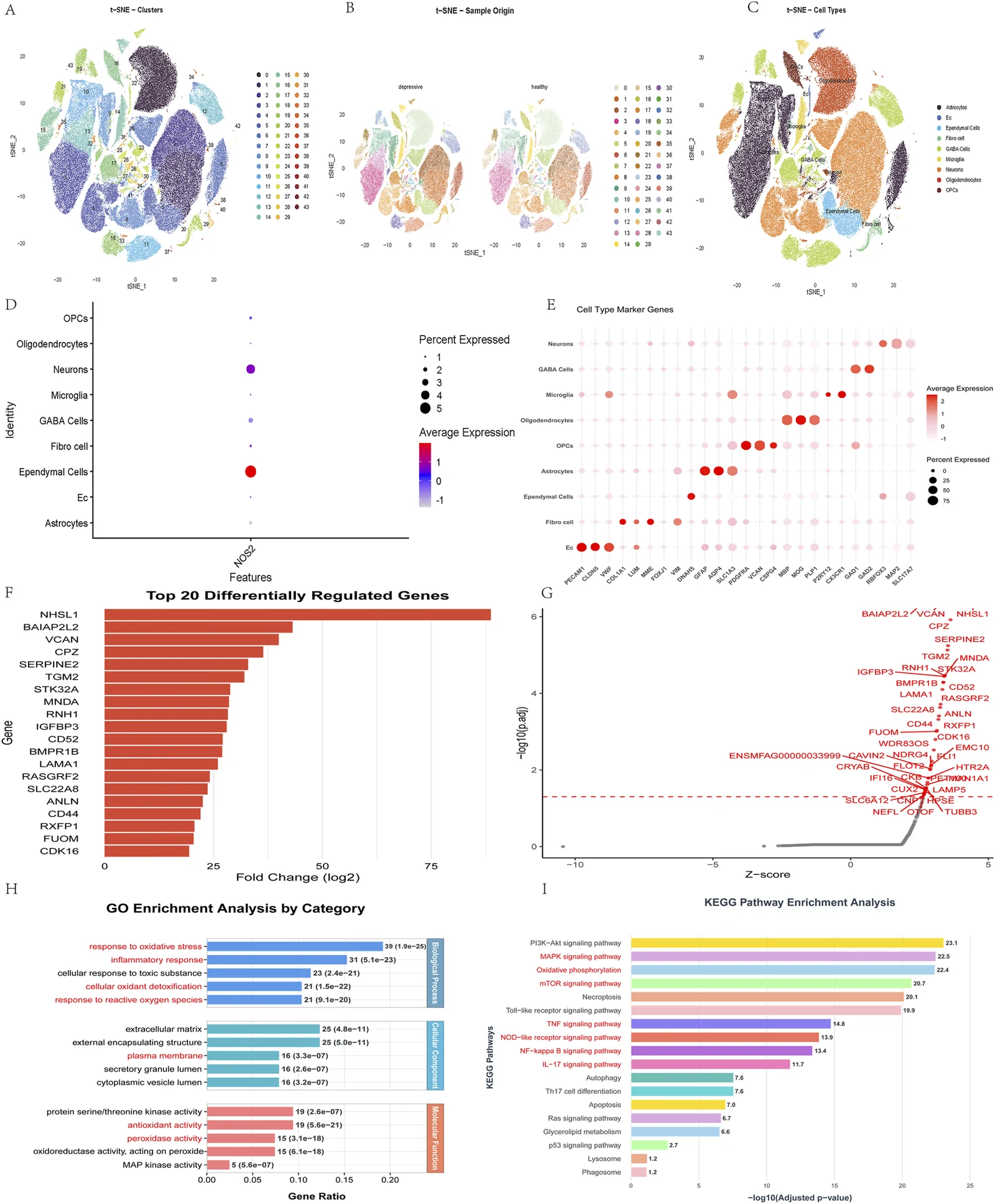
Cellular atlas of the GSE201687 single-nucleus RNA sequencing dataset and downstream analysis of NOS2 virtual perturbation. **(A)** t-SNE clustering plot, with different colors representing 43 cell clusters (0–42). **(B)** t-SNE plot by sample origin, faceted by the depressive and healthy groups, showing that cells from the two groups were well mixed across major cell populations. **(C)** t-SNE cell type annotation plot, with nine major cell types annotated. **(D)** Expression features of NOS2 across different cell types. **(E)** Dot plot of cell type marker genes. **(F)** Bar plot of the top 20 differentially regulated genes after NOS2 virtual perturbation. **(G)** Volcano plot of differentially regulated genes. **(H)** GO enrichment analysis. **(I)** KEGG pathway enrichment analysis.

### Molecular docking results

We performed molecular docking of seven air pollutants with NOS2 and the corresponding heatmap is presented in Figure 7A. The binding energies of the pollutants were as follows: benzene and NOS2, −5.4 kcal/mol; SO_2_ and NOS2, −2.5 kcal/mol; NO and NOS2, −2.9 kcal/mol; CO and NOS2, −1.9 kcal/mol; NO_2_ and NOS2, −3.5 kcal/mol; toluene and NOS2, −5.7 kcal/mol; and O_3_ and NOS2, −3.5 kcal/mol. We investigated seven air pollutants, including benzene, CO, NO, NO_2_, O_3_, and SO_2_. Figures 7B–H illustrates the docking interactions between the seven air pollutants and NOS2. The molecular docking results showed that benzene and toluene exhibited favorable binding with NOS2, whereas the remaining pollutants displayed relatively stable binding. This suggests that all seven air pollutants may be associated with NOS2, especially benzene and toluene.

**FIGURE 7:**
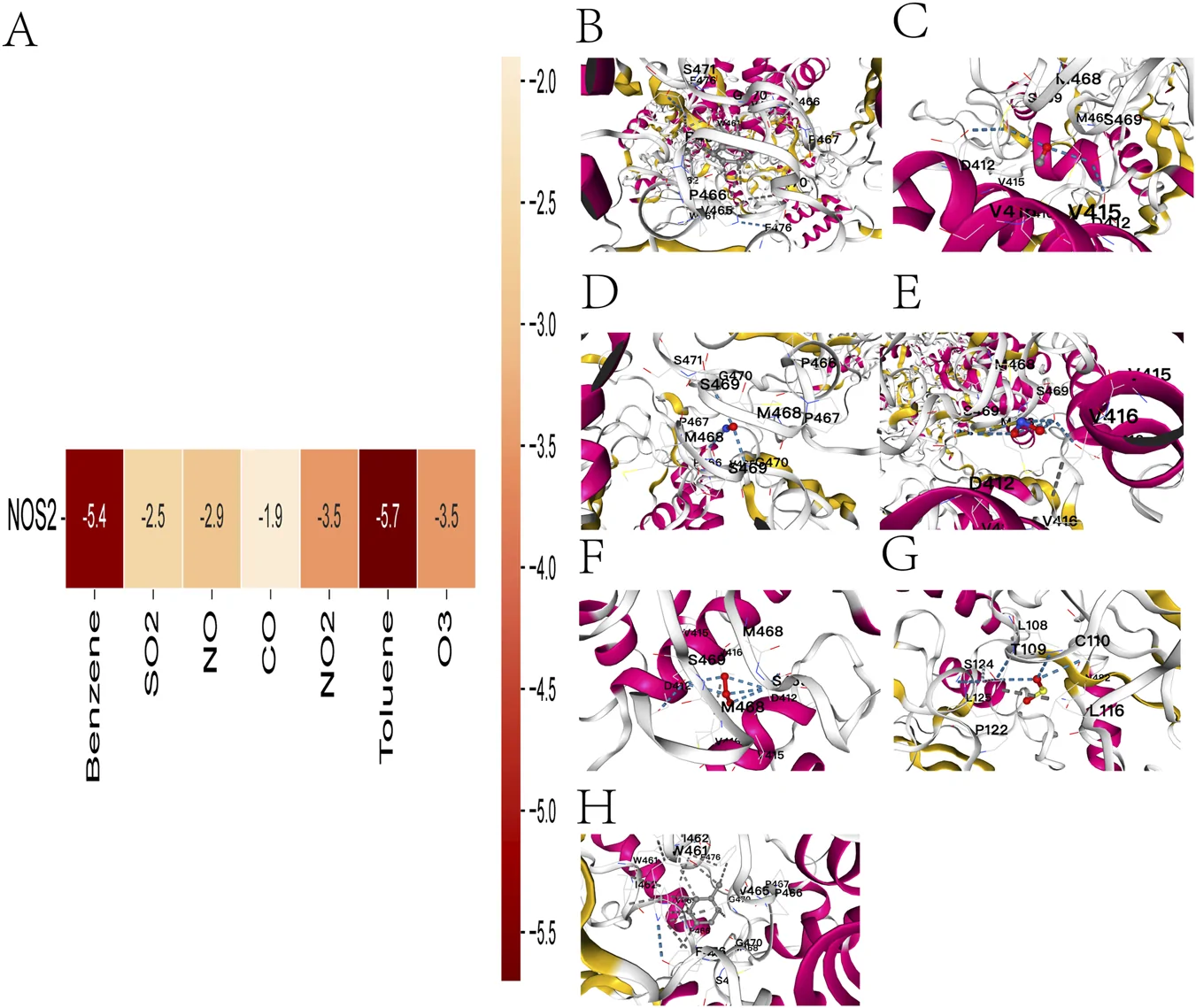
Molecular docking results. **(A)** The heatmap reveals NOS2 and seven air pollutants (benzene, SO _2_, NO, CO, NO _2_, toluene, and O3). **(B)** Visualization of molecular docking between NOS2 and benzene. **(C)** Visualization of molecular docking between NOS2 and CO. **(D)** Visualization of molecular docking between NOS2 and NO. **(E)** Visualization of docking between NOS2 and NO2 molecules. **(F)** Visualization of docking between NOS2 and O3 molecules. **(G)** Visualization of docking between NOS2 and SO2 molecules. **(H)** Visualization of docking between NOS2 and toluene molecules.

### Molecular dynamics simulation results

Subsequently, we performed molecular dynamics simulation experiments. Figure 8A (benzene) shows that the RMSD of free benzene (orange) rose sharply and fluctuated above 10 nm between 10 and 30 ns This indicated that it was highly unstable in the solvent. The RMSD curve of the nitric oxide synthase 2 (NOS2)–benzene complex (green) closely overlapped with that of free NOS2 (blue) and remained stable at approximately 0.2 nm. This indicated that benzene binding did not disrupt the overall structure of NOS2. As shown in Figure 8B (toluene), the RMSD of free toluene (orange) fluctuated between 0.5 and 1.5 nm. The RMSD curve of the NOS2–toluene complex (green) closely overlapped with that of free NOS2 (blue) and remained stable at approximately 0.2 nm. This indicated that toluene binding did not disrupt the structure of NOS2. As shown in Figure 8C, the three curves fluctuated within 1 nm and largely overlapped. This indicated that the amino acid residues of NOS2 were intrinsically rigid and that the addition of benzene or toluene did not significantly alter the local flexibility of individual residues. As indicated in Figure 8D, the three radius of gyration (Rg) curves remained stable between 2.30 and 2.35 nm without significant fluctuations. This suggested that ligand binding did not cause expansion or compression of the overall NOS2 conformation and that the protein remained compact. Figure 8E shows that the three SASA curves remained stable between 210 and 220 nm^2^ without significant fluctuations. This further confirmed that the NOS2 structure was stable and that its stability was not affected by small-molecule binding. As shown in Figures 8F,G (free NOS2), multiple dispersed low-energy basins were observed, indicating the presence of multiple conformational substrates of free NOS2. Figures 8H,I (NOS2–benzene complex) show that two relatively dispersed lowest energy clusters were formed, suggesting that the conformational stability of the NOS2–benzene complex was relatively poor. As shown in Figures 8J,K (NOS2–toluene complex), a single, concentrated dark-blue low-energy basin was formed, indicating that the NOS2–toluene complex was structurally stable. Figures 8L,M show that the average binding free energies between the NOS2 protein and benzene and toluene were −9.27 and −12.75 kcal mol^-1^, respectively. This indicated that the binding affinity of the NOS2 protein was slightly weaker for benzene compared to toluene.

**FIGURE 8:**
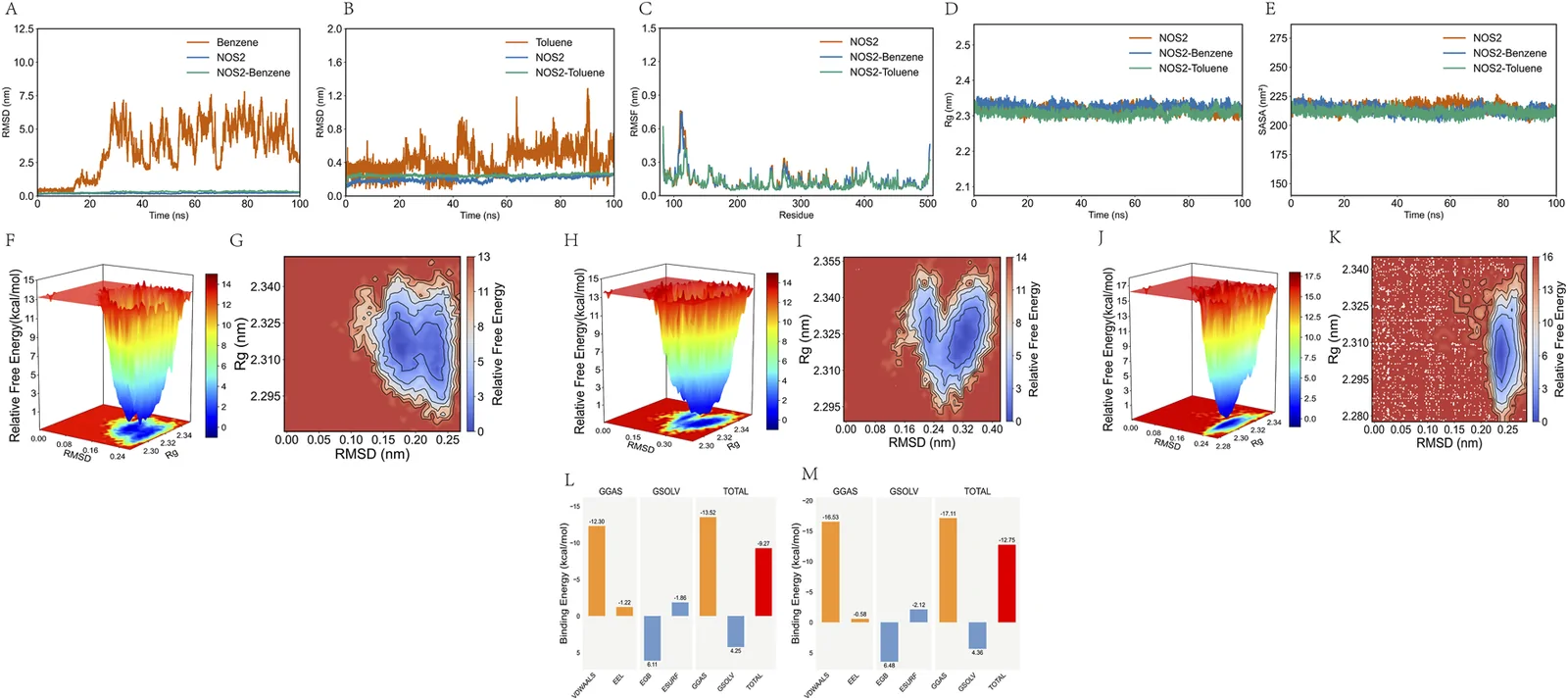
Molecular dynamics simulation results (Benzene and toluene. **(A, B)** RMSD Curve of NOS2 Protein Complexed with Benzene and Toluene. **(C)** RMSF curve of NOS2 protein complexed with Benzene and Toluene. **(D)** Rg curve of NOS2 protein complexed with Benzene and Toluene. **(E)** SASA curve of the NOS2 protein complexed with Benzene and Toluene. **(F-K)** Free Energy Distribution Map of NOS2 Protein Complexes with Benzene and Toluene. **(L, M)** Average binding free energy of NOS2 protein with Benzene and Toluene. (VDWAALS, EEL, EGB, ESURF, GGAS, GSOLV, and TOTAL represent van der Waals forces, electrostatic energy, polar solvation energy, nonpolar solvation energy, molecular mechanics term, solvation energy term, and average binding free energy, respectively).

### Toluene exposure induces inflammation and oxidative stress in BV2 cells, which may be associated with NOS2

The CCK8 assay results are shown in Figures 9A,B. Treatment with 20 μM toluene for 24 and 48 h did not significantly affect BV2 cell viability. Treatment with 50 μM toluene did not show a significant effect at 24 h; however, decreased cell viability at 48 h (P = 0.0185); treatment with 100 μM toluene significantly reduced cell viability at 24 h (P = 0.0023) and 48 h (P < 0.0001). The OD values are shown in Figure 9C. The LDH assay results are shown in Figures 9D,E. Treatment with 20 μM toluene for 24 or 48 h did not significantly alter cellular LDH levels; treatment with 50 μM toluene did not significantly change cellular LDH levels at 24 h; however, significantly increased LDH at 48 h (P = 0.032); treatment with 100 μM toluene significantly increased cellular LDH at 24 h (P < 0.0001) and 48 h (P < 0.0001). Subsequently, we selected the 24-h treatments with 50 μM and 100 μM toluene for cell assays. The western blotting results in Figures 9F,G show that treatment with 50 μM toluene did not significantly alter NOS2 protein expression in the BV2 cells; treatment with 100 μM toluene significantly increased the level of NOS2 protein in the BV2 cells (P < 0.0001). Compared with the 50 μM toluene group, the 100 μM toluene group showed significantly higher NOS2 protein levels (P = 0.0004). As shown in Figure 9H, treatment with 50 μM toluene did not significantly alter the NOS2 mRNA levels in the BV2 cells. Compared with the control group and the 50 μM toluene group, treatment with 100 μM toluene significantly increased NOS2 mRNA levels in the BV2 cells (P < 0.0001). The nitrite detection results are shown in Figure 9I. Treatment with 50 μM toluene did not significant change the cellular nitrite levels, whereas treatment with 100 μM toluene significantly increased cellular nitrite levels (P < 0.0001). Figure 9J shows that treatment with 50 μM toluene did not significantly alter the mRNA expression levels of TNF-α, IL-1β, IL-6, and NF-κB p65 in the BV2 cells; however, treatment with 100 μM toluene significantly increased the mRNA levels of TNF-α (P < 0.0001), IL-1β ((P < 0.0001), IL-6 (P = 0.0050), and NF-κB p65 (P < 0.0001) in the BV2 cells. The ROS detection results are shown in 9K and 9L. Treatment with 50 μM toluene did not significantly change the cellular ROS levels, whereas treatment with 100 μM toluene significantly increased cellular ROS levels (P < 0.0001). The immunofluorescence results are shown in Figures 9M,N. Treatment with 50 μM toluene did not significantly alter the cellular 3-nitrotyrosine levels; however, treatment with 100 μM toluene significantly increased the cellular 3-nitrotyrosine levels (P = 0.0064).

**FIGURE 9:**
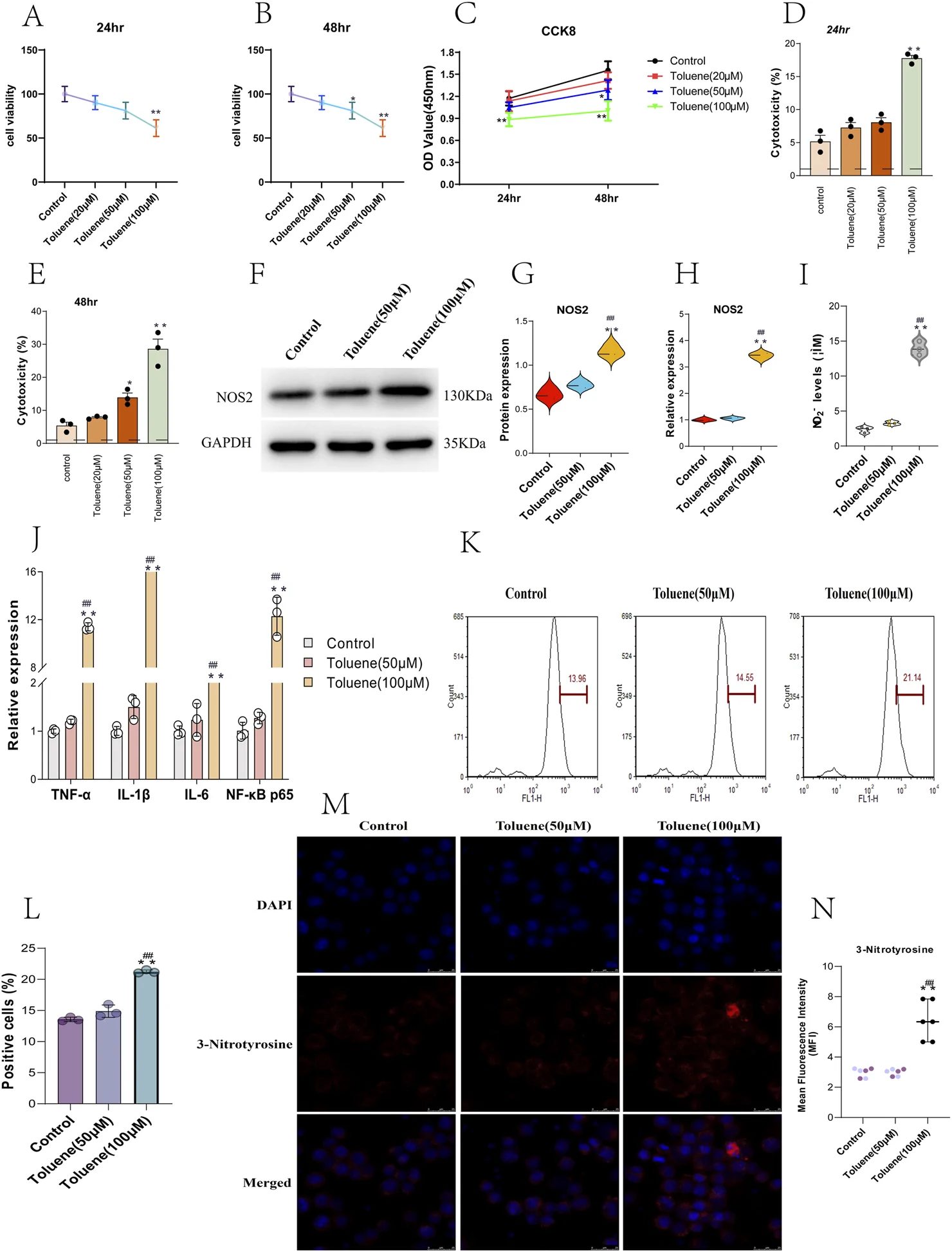
Toluene exposure induces inflammation and oxidative stress in BV2 cells, with possible involvement of NOS2. **(A)** CCK-8 assay of BV2 cell viability after exposure to various concentrations of toluene for 24 h. **(B)** CCK-8 assay of BV2 cell viability after exposure to various concentrations of toluene for 48 h. **(C)** Optical density (OD) values in each group. **(D)** Lactate dehydrogenase (LDH) assay of the cytotoxic effects of various concentrations of toluene on BV2 cells after 24 h. **(E)** LDH assay of the cytotoxic effects of various concentrations of toluene on BV2 cells after 48 h. **(F)** Protein expression of NOS2 in BV2 cells after 24 h of treatment with various concentrations of toluene. **(G)** Quantification of NOS2 protein expression in BV2 cells after 24 h of treatment with various concentrations of toluene. **(H)** Quantification of NOS2 mRNA levels in BV2 cells after 24 h of treatment with various concentrations of toluene. **(I)** Nitrite levels in BV2 cells after 24 h of treatment with various concentrations of toluene. **(J)** mRNA levels of TNF-α, IL-1β, IL-6, and NF-κB p65 in BV2 cells after 24 h of treatment with various concentrations of toluene. **(K)** Flow cytometric analysis of ROS levels in BV2 cells after 24 h of treatment with various concentrations of toluene. **(L)** Quantification of ROS levels in BV2 cells after 24 h of treatment with various concentrations of toluene. **(M)** Immunofluorescence staining for 3-nitrotyrosine in BV2 cells after 24 h of toluene treatment. **(N)** Quantification of 3-nitrotyrosine immunofluorescence in BV2 cells after 24 h of toluene treatment. Data are presented as mean ± SD of three technical replicates (n = 3). *P < 0.05, **P < 0.01 vs. the control group; #P < 0.05, ##P < 0.01 vs. the 50 μM toluene group.

## Discussion

Environmental risks—including air, water, and noise pollution, as well as biological materials and microbial contamination—substantially affect human health. The relationship between environmental risks and health—particularly the association between air pollution and chronic diseases—has been extensively studied (Briggs, 2003). The public health impact of environmental pollution primarily encompasses adverse health outcomes, public health expenditure, and health inequalities. Air pollution contributes more substantially to global disease incidence and mortality than all other environmental risk factors combined (Al-Kindi et al., 2020). Exposure to air pollution is associated with acute respiratory conditions (such as asthma) and chronic cardiovascular disorders, and prolonged exposure increases chronic disease incidence. High concentrations of air pollutants induce inflammation in the respiratory and circulatory systems or trigger allergic reactions (Raza et al., 2018), thereby contributing to respiratory and cardiovascular diseases and increased disease mortality (Yu et al., 2020). Several studies have demonstrated a significant link between air pollution and various chronic diseases (Hu and He, 2022). Previous reports have also shown association between air pollution and depression (Zhang et al., 2017).

Depression is a common, severe chronic mental disorder characterized by low mood, loss of interest, cognitive slowing, sleep disturbances, executive dysfunction, and social impairment. In severe cases, depression may lead to suicidal tendencies (Guo et al., 2020). Depression is highly prevalent, with women more commonly affected than men. It can occur at various life stages, including childhood, adolescence, and old age, with the highest incidence during adulthood (GBD 2019 Mental Disorders Collaborators, 2019). Current statistics indicate that 13%–20% of individuals experience depression, whereas the lifetime prevalence is 6.1%–28.2% (Peng et al., 2015; Vandeleur et al., 2017). Global studies indicate that Southeast Asia and the Western Pacific regions account for 50% of worldwide depression cases; Afghanistan exhibiting the highest prevalence with over 20% of its population experiencing depression (World Health Organization, 2017). The global incidence of depression increased by 49.86% from 1990 to 2017 (Liu et al., 2020). Moreover, the sudden global public health event of the COVID-19 pandemic in 2020 accelerated and exacerbated various mental symptoms, especially depression and anxiety disorders (Yang et al., 2020). Although depression has been extensively studied in recent years, its exact causes remain unclear. Current mechanisms include neurotransmitter receptor abnormalities, neurodegeneration, endocrine factors, inflammatory cytokines, epigenetic regulation, and brain-derived neurotrophic factors. Depression is influenced by regulatory factors such as circadian rhythm disruption, light exposure effects, oxidative stress, and genetic factors. Recent reviews, meta-analyses, and research utilizing databases such as NHANES and CHARLS, have increasingly focused on the link between air pollution and depression. However, these studies have largely investigated correlations between air pollutants and depression without specifically analyzing the mechanisms by which air pollution affects the onset of depression. In this study, we integrated network toxicology analysis with depression-related transcriptomic data from the GEO database, and performed weighted coexpression network analysis, machine learning, molecular docking, molecular dynamics simulations, Mendelian randomization, single-cell virtual perturbation, and *in vitro* cellular experiments to investigate the potential mechanism by which air pollution induces depression.

In this discussion, we distinguish four levels of evidence: (i) associative evidence from network toxicology and database intersections; (ii) computational predictions from machine learning, molecular docking, molecular dynamics simulations, and single-cell virtual perturbation; (iii) genetic causal inference from MR; and (iv) biological consequences and mechanistic analysis from *in vitro* experiments.

In this study, we identified nine common genes related with air pollution and depression: NOS2, HRH4, PNMT, GRM2, PTGES, ABCG2, CA3, F2, and CA7. Subsequently, through ensemble machine learning, we identified five potentially prognostic genes: CA7 (AUC = 0.621), F2 (AUC = 0.582), GRM2 (AUC = 0.647), NOS2 (AUC = 0.659), and PTGES (AUC = 0.503). Statistical analysis of these five genes demonstrated that NOS2 was significantly upregulated in depression (P < 0.05). The other four genes did not show statistical significance; AUC value was highest for NOS2. On this basis, we prioritized NOS2 as a candidate hub gene for subsequent analyses. Notably, these machine learning findings are computational and associative in nature and do not establish causality. The nitric oxide synthase 2 (NOS2) gene is located on human chromosome 17q11.2. This gene encodes a protein belonging to the flavoprotein family. It is also known as iNOS, NOS, or HEP-NOS. Its OMIM number in the gene databases is 163,730; its Ensembl number is ENSG00000007171; and UniProt number for the NOS2 protein is P35228. Elevated levels of proinflammatory cytokines (IL-6, TNF-α) in the peripheral blood of patients with depression activate microglia in the brain and induce iNOS expression (Miller et al., 2009). In chronically stressed mice, hippocampal iNOS expression increases 2–3-fold and is accompanied by microglial activation; depressive-like behavior is significantly alleviated in NOS2 knockout (KO) mice (Wohleb, 2016). Furthermore, iNOS enhances neuroinflammation by activating the TLR4/MyD88 pathway (Frank, 2019). A meta-analysis indicated that the A allele (Leu608) is associated with increased iNOS activity and an elevated risk of depression (OR = 1.34, 95% CI: 1.12–1.60) (Galecki, 2011). We conducted MR and observed that NOS2 increased the risk of depression to investigate the relationship between NOS2 and depression. MR analysis provides genetic-level causal inference. However, since MR relies on assumptions regarding instrument validity, the downstream pathways through which NOS2 influences depression were not directly tested here.

Based on previous studies (Li et al., 2025), we focused on seven air pollutants: benzene, SO_2_, NO, CO, NO_2_, toluene, and O_3_. Molecular docking and molecular dynamics simulation experiments predicted that all seven pollutants potentially interacted with NOS2. Among these, benzene and toluene showed the highest predicted binding affinities and the toluene–NOS2 complex appeared more stable. These docking and dynamics results are computational predictions rather than experimental binding measurements and should not be overinterpreted as evidence that all seven pollutants bind strongly to NOS2. Toluene exposure significantly increases hippocampal NO levels in the high-dose exposure group compared with the control group. Hippocampal NOS activity was significantly higher in rats exposed to high doses than in controls, with a notable correlation between the two. Toluene increases glutamate levels in the brain tissue, leading to elevated NOS activity. This increases NO production, leading to the generation of large amounts of free radicals, subsequently causing brain tissue damage (Yan, 2004). Studies have shown that long-term low-level SO_2_ exposure enhances astrocyte and microglia activation in the rat cerebral cortex, resulting in elevated mRNA expression of inflammatory markers such as TNF-α, IL-1β, iNOS, and COX-2. These findings suggest that SO_2_ exposure alters cellular signaling and nuclear transcription through inflammatory responses, ultimately leading to neurofunctional damage (Yao and Sang, 2015).

Computational simulation analyses in this study suggest that NOS2 may act as a regulatory factor linking air pollutants and depression. Single-cell virtual perturbation of NOS2 significantly altered pathways related to inflammatory responses and oxidative stress. As an *in silico* network perturbation approach, this provides mechanistic hypothesis rather than direct mechanistic proof. The cytokine hypothesis, also known as the “inflammatory factor hypothesis,” suggests that inflammatory responses affect neurogenesis in the brain and interfere with the normal functions of nerve cells, leading to depressive-like behaviors (Tang et al., 2017). Studies (Beurel et al., 2020) have shown that levels of proinflammatory cytokines are higher in patients with depression than in healthy individuals; moreover, and administration of exogenous proinflammatory cytokines to healthy individuals can also induce depression. Therefore, inhibiting neuroinflammation is important for treating depression. In a depressive state, hyperactivated microglia release inflammatory factors, accelerate neuroinflammation, induce microglial dysfunction, and damage normal neurological functions, thereby worsening depressive symptoms (Kwon and Koh, 2020). Therefore, hyperactivation of mouse microglia (BV2) is a potential pathogenic mechanism of depression. Oxidative stress plays an important biological role in the pathogenesis of depression. Oxidative stress refers to a pathological state in which the production of ROS exceeds the clearance capacity of the intracellular antioxidant system, leading to damage to cellular structure and function (Migdal and Serres, 2011). The levels of oxidative stress biomarkers in the plasma and brain tissue are significantly altered and abnormal in patients with depression (Edem et al., 2023). Oxidative stress directly impairs mitochondrial function and can also participate in disease progression through multiple pathways, including regulation of apoptosis and neuroinflammation. As the increase in NOS2-induced inflammatory responses may contribute to depression and toluene exposure causes oxidative stress by increasing NO levels, we used BV2 cells to observe inflammatory and oxidative stress responses following toluene exposure. In the *in vitro* BV2 cell experiments, exposure to 100 μM toluene significantly reduced cell viability and was accompanied by increased NOS2 protein and mRNA levels, as well as increased mRNA levels of TNF-α, IL-1β, IL-6, and NF-κB p65. The increase in NOS2 level was accompanied by elevated ROS and nitrite levels (stable metabolites of NO), subsequently leading to increased expression of 3-nitrotyrosine. 3-Nitrotyrosine (3-NT) is a product of tyrosine nitration mediated by nitrogen dioxide and peroxynitrite anions and is considered as a NO-dependent biomarker of nitrosative stress. Studies have found that 3-NT levels are significantly elevated in patients with depression (Baek and Park, 2013), middle-aged women at risk of depression (Pasquali et al., 2018), adolescents with depression (Katrenčíková et al., 2021), and patients with post-stroke depression (Cichoń et al., 2015). These cellular findings are consistent with the computational predictions and support a plausible NOS2-related inflammatory and oxidative stress response. However, since these effects were observed under cytotoxic exposure conditions, cytotoxicity-related confounding cannot be excluded, and these data do not establish that NOS2 causally mediates depression.

The main strength of this study is that we used a multilevel experimental framework to investigate the specific molecular mechanisms potentially linking air pollution to depression. Depression-related targets were identified through network-weighted coexpression analysis using the GEO database and intersected with the air pollution targets to identify air pollution-related depression targets. Ensemble machine learning was then used for target screening and validation, followed by further computational evaluation using molecular docking, molecular dynamics simulations, and MR. Single-cell virtual perturbation was used to generate mechanistic hypotheses, and *in vitro* cellular experiments were conducted to assess whether biological results were consistent with the computational predictions and corresponding mechanisms. Importantly, these different evidence levels—association, computational prediction, genetic causal inference, and biological assays—should be interpreted separately rather than as a single causal chain.

This study has several limitations. First, the current conclusions are primarily based on computational simulations, bioinformatics analyses, and cellular experiments. Although these findings suggest potential mechanistic pathways (such as NOS2-mediated neuroinflammatory and oxidative stress responses), they have not been validated in animal models. Further *in vivo* experiments, such as measuring changes in the expression of relevant genes and proteins and verifying signaling pathway activities, are necessary to empirically confirm the causal relationship. Second, although the public databases used in this study (e.g., GEO) are valuable, their data originate from different research teams, technical platforms, and experimental designs. This may introduce batch effects, sample selection bias, or technical inconsistencies, all of which may affect the stability and generalizability of the gene expression analysis results. Future validation should incorporate multicenter, independently collected datasets with standardized protocols. Third, MR can provide genetic causal inference; however, relies on strict assumptions, including the validity of instrumental variables and the absence of horizontal pleiotropy; moreover, colocalization analysis was not performed. Therefore, MR findings should be interpreted with caution. Fourth, single-cell virtual perturbation and molecular docking/dynamics are computational approaches that generate predictions and hypotheses rather than direct experimental evidence. Fifth, the cellular effects observed in this study were obtained under exposure conditions associated with cytotoxicity, because 100 μM toluene significantly reduced cell viability. Therefore, the observed inflammatory and oxidative stress responses may be partially influenced by cytotoxicity, and the cellular results should be regarded as providing a biological basis for further experimentation rather than definitive mechanistic proof. Future studies should use subcytotoxic dose ranges, NOS2 inhibition or knockdown, and *in vivo* models to more rigorously test whether NOS2 causally mediates the link between air pollutant exposure and depression. Sixth, molecular docking, SwissTargetPrediction, TargetNet, and SEA are primarily structure-based tools developed for small organic molecules, and their applicability to simple gaseous molecules such as CO, NO, NO_2_, SO_2_, and O_3_ is limited. These predictions should therefore be regarded as exploratory and hypothesis-generating. In molecular dynamics simulations, we did not perform protein–ligand distance and time-dependent interaction or contact occupancy analyses. Therefore, these results should be interpreted with caution and require further confirmation.

## Conclusion

This study employed a comprehensive computational strategy integrating network toxicology, machine learning, molecular docking, molecular dynamics simulations, Mendelian randomization, and single-cell virtual perturbation to explore the potential link between air pollutants and depression. The results suggest that NOS2 may serve as a key mediator connecting air pollutant exposure to the development of depression, with its regulatory mechanism potentially involving neuroinflammatory responses and oxidative stress, as illustrated in Figure 10. Specifically, after inhalation of toxic gases such as toluene and benzene, these substances cross the blood–brain barrier, activate microglia in the brain, and trigger inflammatory and oxidative stress responses. In microglia, NOS2 is expressed, and the resulting nitric oxide (NO) promotes alterations in the transcript abundance of NF-κB p65, facilitating the synthesis of pro-inflammatory cytokines including TNF-α, IL-1β, and IL-6, thereby leading to neuroinflammation. Concurrently, activated microglia generate large amounts of reactive oxygen species (ROS). ROS combines with NO to produce peroxynitrite, which undergoes activation and decomposition to generate more reactive free radicals, mediating the nitration of tyrosine residues and ultimately forming 3-nitrotyrosine, resulting in protein dysfunction, cellular structural damage, and oxidative/nitrosative stress, ultimately causing neuronal dysfunction and synaptic damage. Neuroinflammation and synaptic damage arising from neuronal dysfunction may jointly contribute to the onset of depression. These findings provide valuable theoretical insights into the relationship between air pollutants and depression. Future studies should thoroughly investigate the functional mechanism of the NOS2 gene and its potential clinical applications.

**FIGURE 10:**
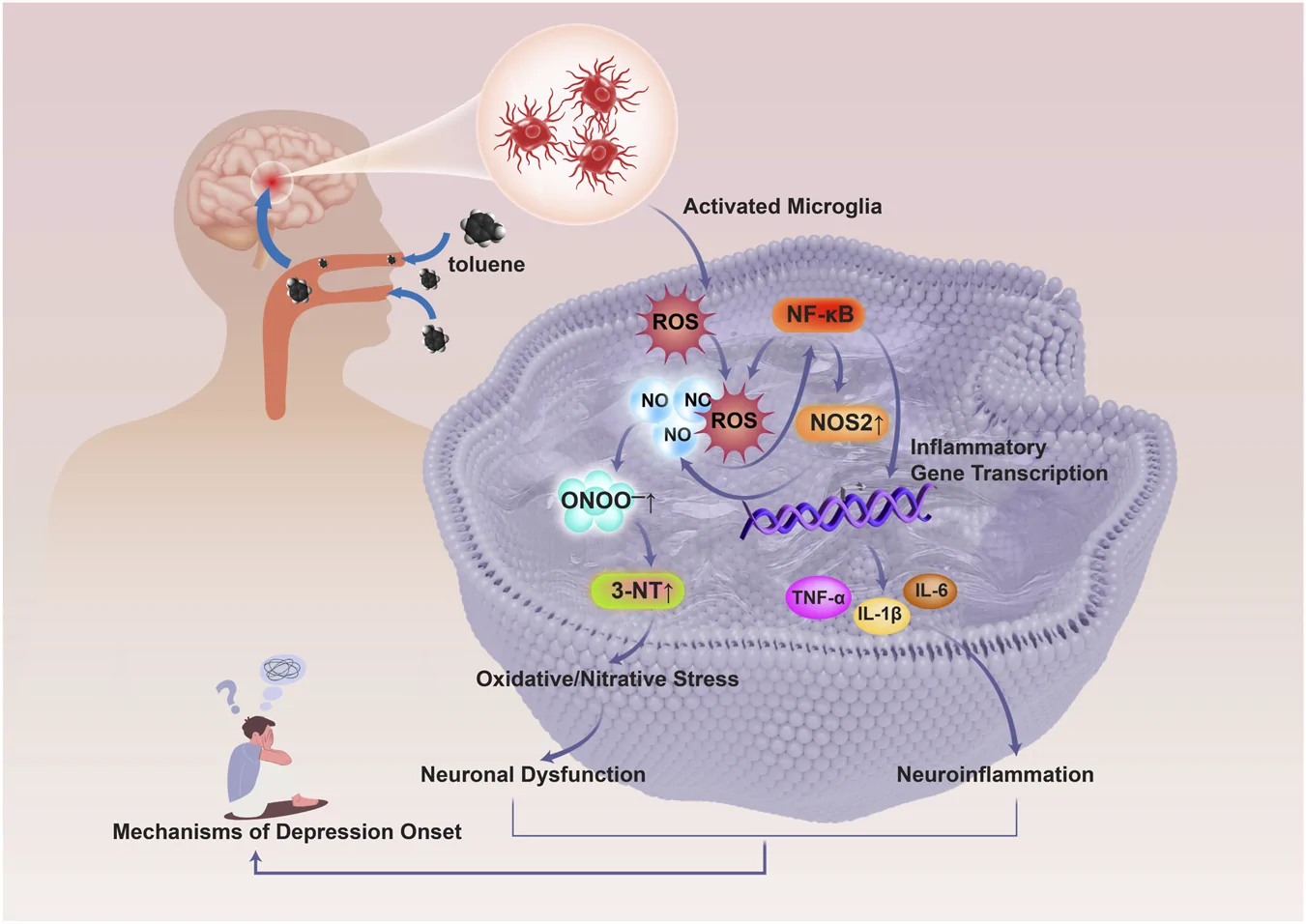
The potential link between environmental pollutants (toluene) and depression may involve NOS2-related neuroinflammation and oxidative stress(proposed working model). Nitric oxide synthase 2(NOS2), Reactive oxygen species(ROS), Nitric oxide(NO),Nuclear Factor kappa B(NF-κB), 3-Nitrotyrosine (3-NT), Tumor Necrosis Factor alpha(TNF-α), Interleukin-6(IL-6), Interleukin-1 beta(IL-1B).

## Data Availability

The original contributions presented in the study are included in the article/Supplementary Material, further inquiries can be directed to the corresponding authors.

## Appendix A Supporting information

Air pollution target as Supplementary Table S1, depression target as Supplementary Table S2, SNPs information for Mendelian randomization analysis as Supplementary Table S3.
